# Supplementary material for: CD8+ T-cell responses towards conserved influenza B virus epitopes across anatomical sites and age
Source: Nat Commun. 2024 Apr 29;15:3387. doi: 10.1038/s41467-024-47576-y (PMC11059233; doi:10.1038/s41467-024-47576-y)
Supplement: Supplementary file 1 — Supplementary Information [file 41467_2024_47576_MOESM1_ESM.pdf]

SUPPLEMENTARY FILES

Tejas Menon[1,*], Patricia T. Illing[2,*], Priyanka Chaurasia[2,*], Hayley A. McQuilten[1], Chloe Shepherd[2], Louise C. Rowntree[1], Jan Petersen[2], Dene R. Littler[2], Grace Khuu[2], Ziyi Huang[2], Lilith F Allen[1], Steve Rockman[1,3], Jane Crowe[4], Katie L. Flanagan[5,6,7], Linda M. Wakim[1], Thi H. O. Nguyen[1], Nicole A. Mifsud[2], Jamie Rossjohn[2,8,#], Anthony W. Purcell[2,#], Carolien E van de Sandt[1,9,#] and Katherine Kedzierska[1,#]

[1]Department of Microbiology and Immunology, University of Melbourne, at the Peter Doherty Institute for Infection and Immunity, Parkville, Australia.
[2]Infection and Immunity Program & Department of Biochemistry and Molecular Biology, Biomedicine Discovery Institute, Monash University, Clayton, Australia.
[3]Seqirus, Parkville, VIC, Australia.
[4]Deepdene Surgery, Deepdene, Victoria, Australia
[5]Tasmanian Vaccine Trial Centre, Launceston General Hospital, Launceston, TAS, Australia.
[6]School of Health Sciences and School of Medicine, University of Tasmania, Launceston, TAS, Australia.
[7]School of Health and Biomedical Science, RMIT University, Melbourne, VIC, Australia.
[8]Institute of Infection and Immunity, Cardiff University School of Medicine, Cardiff, UK.
[9]Department of Hematopoiesis, Sanquin Research, Amsterdam, The Netherlands, and Amsterdam UMC location University of Amsterdam, Landsteiner Laboratory; Amsterdam, The Netherlands.
[*]These authors contributed equally
[#]These authors jointly supervised the work

Correspondence: kkedz@unimelb.edu.au

Supplementary Fig. 1 Gating strategy for human ICS.
Supplementary Fig. 2 Frequencies expanded HLA-I-peptide-specific CD8+ T-cell populations.
Supplementary Fig. 3 *Ex vivo* characterisation of IBV-specific CD8$^+$ T-cells across age groups.
Supplementary Fig. 4 IBV-specific CD8$^+$ T-cells across human tissues.
Supplementary Fig. 5 Analysis of TCR repertoire.
Supplementary Fig. 6 Gibbs cluster analysis of IBV peptides.

Supplementary Table 1. Selected HLA-B*07:02, -B*08:01 and B*35:01 restricted IBV peptides.
Supplementary Table 2. Study donor demographics.
Supplementary Table 3. Immunogenic B7-I, B8-I and B35-I pools.
Supplementary Table 4. Data collection and refinement statistics.
Supplementary Table 5. Expanded clonotypes.
Supplementary Table 6. Clonotype counts of B7/NP$_{30-38.}$
Supplementary Table 7. Clonotype counts of B8/NP$_{30-38.}$
Supplementary Table 8. Clonotype counts of B7/NS1$_{196-206.}$
Supplementary Table 9. Clonotype counts of B8/NP$_{92-99.}$
Supplementary Table 10. TCR sequencing primers

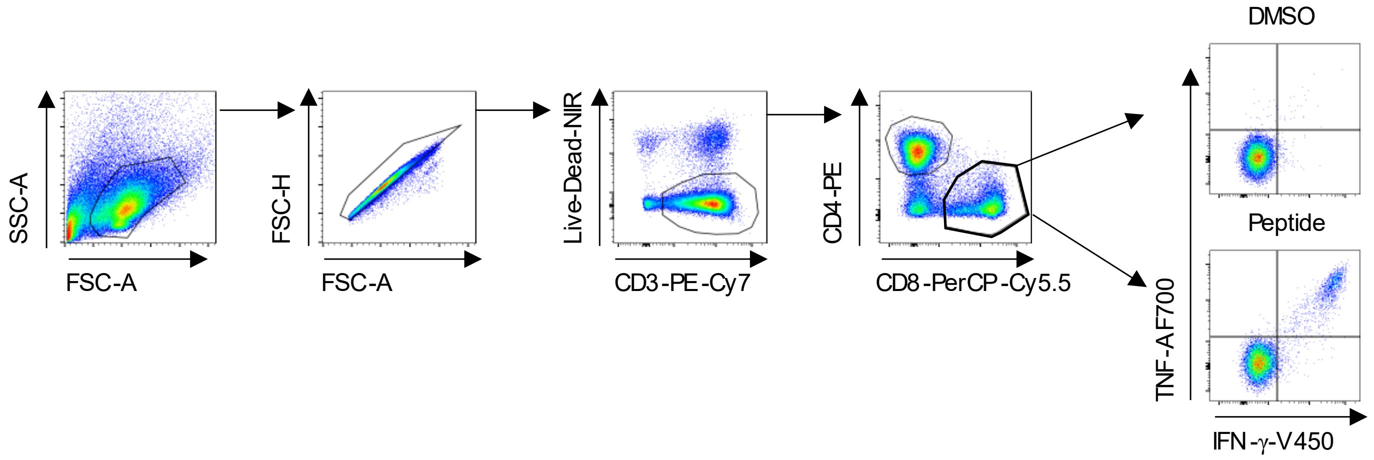

Supplementary Fig. 1. Gating strategy for human ICS. IFN-γ and TNF producing CD8$^+$ T-cells were identified by first gating on CD3$^+$ live lymphocytes.

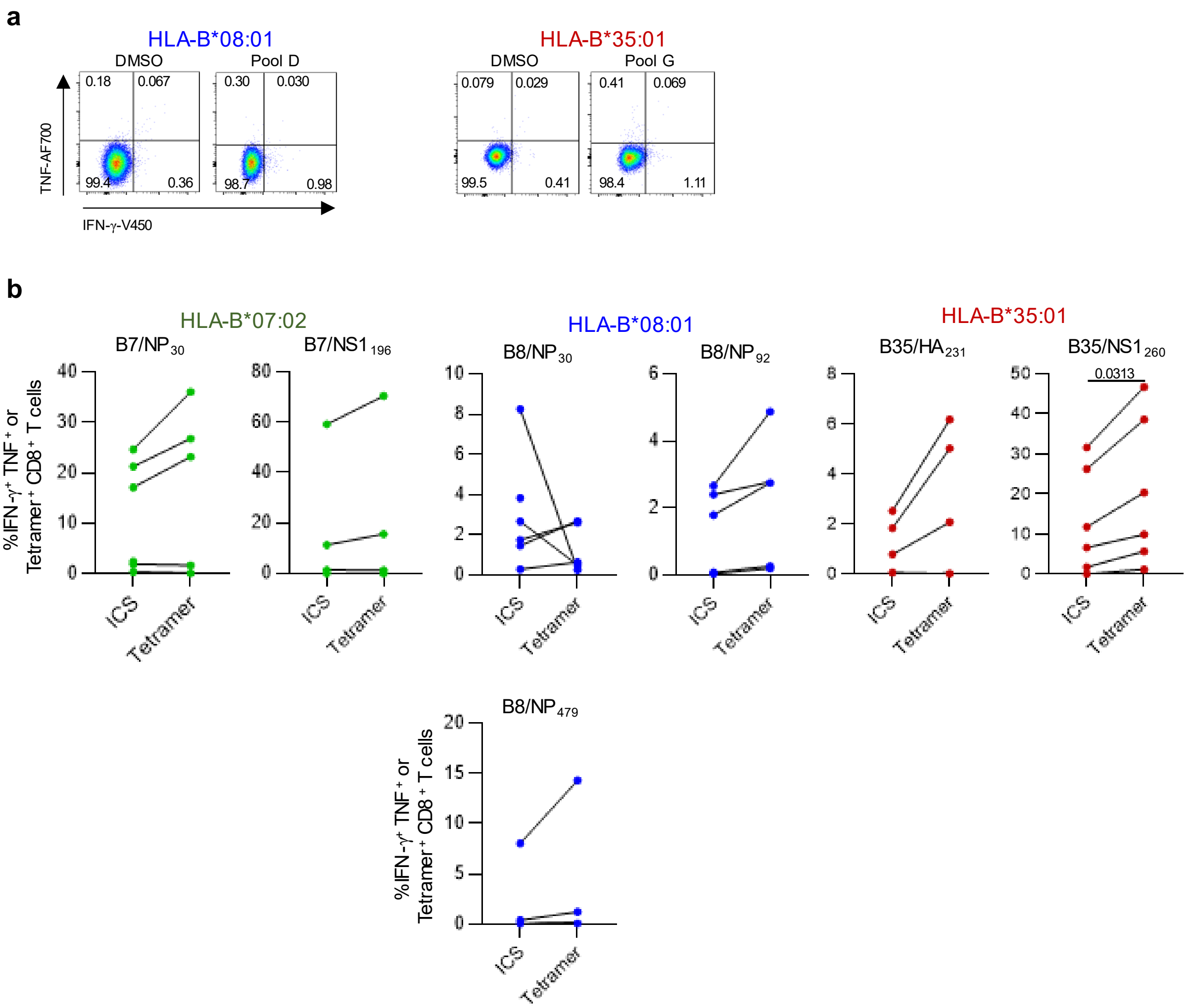

Supplementary Fig. 2. Frequencies expanded HLA-I-peptide-specific CD8⁺ T-cell populations. (a) Representative FACS plots of IFN-γ⁺TNF⁺ CD8⁺ T-cells restimulated with peptide pools D or G on day 10. **(b)** Comparison of IFN-γ⁺TNF⁺ and tetramer⁺ frequencies in IBV-specific CD8⁺ T-cells within day 12 expanded pool-specific CD8⁺ T-cells (n=4-6 donors). Statistical significance was determined with the Wilcoxon matched-pairs signed rank test. Source data are provided as a Source Data file.

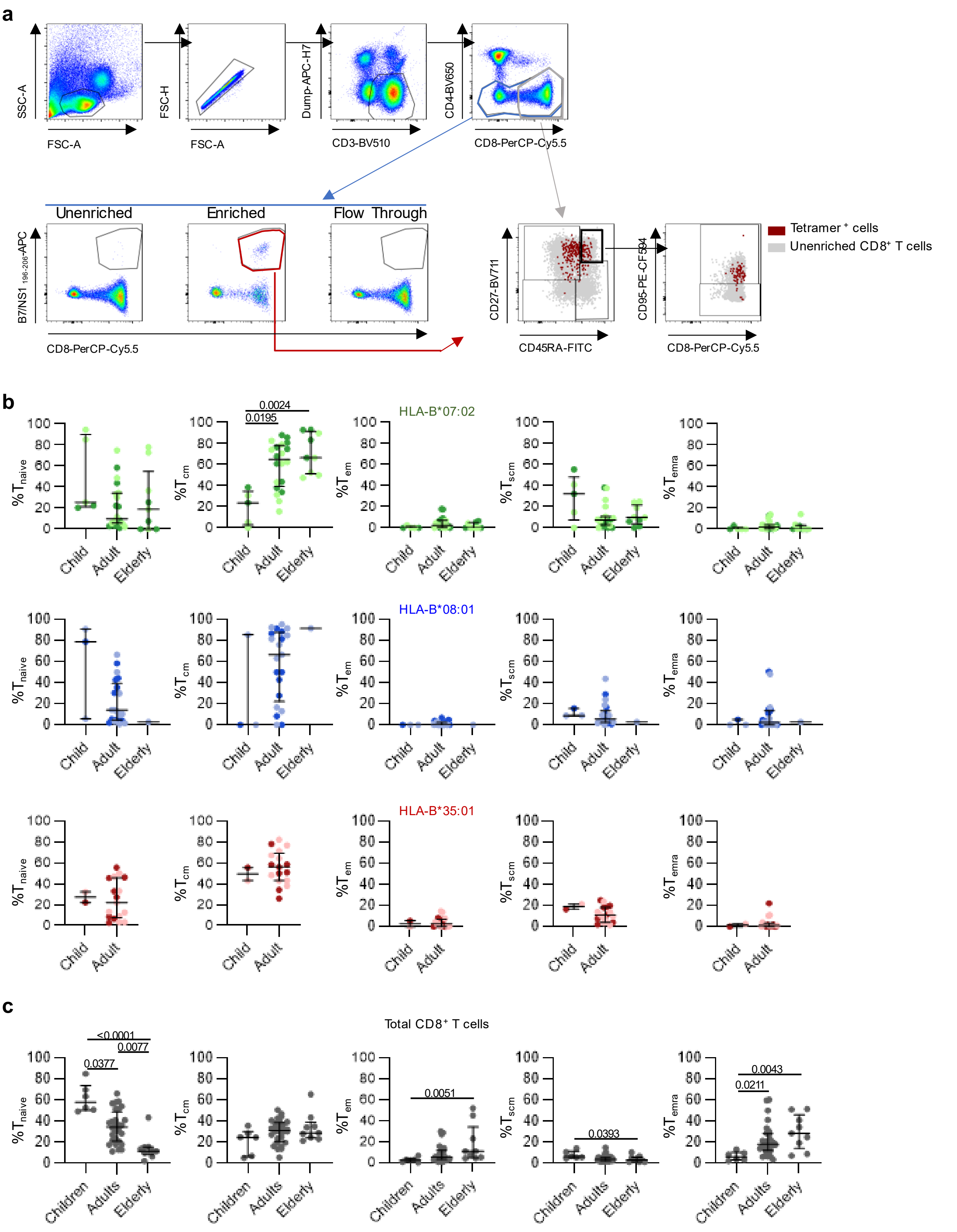

Supplementary Fig. 3. *Ex vivo* characterisation of IBV-specific CD8⁺ T-cells across age groups. (a) Representative FACS gating strategy used to characterize the total CD8⁺ T-cell population and IBV-specific CD8⁺ T-cells. The second line indicates unenriched, enriched and flowthrough fractions of the TAME assay. The unenriched fraction was used to characterize the frequency and phenotype of the total

$CD8^+$ T-cell population (grey gate and cell populations), whereas the IBV-specific $CD8^+$ T-cells of the enriched fraction were used to characterize the frequency and phenotype of the IBV-specific $CD8^+$ T-cells (red gate and cell populations). Naïve/memory T-cell subsets were identified as T_{cm} ($CD27^+CD45RA^-$) cells, T_{eff} ($CD27^-CD45RA^-$), T_{emra} ($CD27^-CD45RA^+$), $T_{naïve}$ ($CD27^+CD45RA^+CD95^-$) and T_{scm} ($CD27^+CD45RA^+CD95^+$) cells. **(b)** IBV/HLA-B*07:02 tetramers (n=18 donors), IBV/HLA-B*08:01 tetramers (n=14 donors) and IBV/HLA-B*35:01 tetramers (n=10 donors). **(c)** Proportions of memory phenotypes of total $CD8^+$ T-cells in children (n=6), adults (n=28) and elderly donors (n=9). **(b-c)** Statistical significance was determined using a two tailed Kruskal-Wallis with Dunn's test for multiple comparisons (HLA-B*07:02, HLA-B*08:01 and Total $CD8^+$ T cells) or **(b)** Mann-Whitney U test (HLA-B*35:01). Source data are provided as a Source Data file.

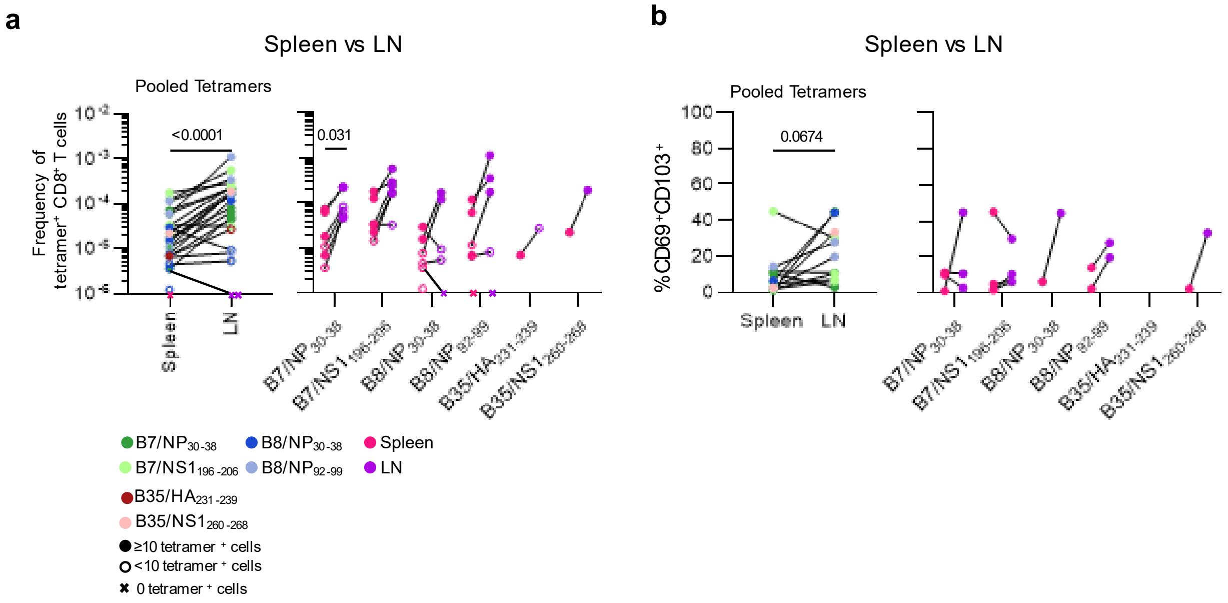

Supplementary Fig. 4. IBV-specific CD8⁺ T-cells across human tissues. (a) Frequency of IBV-specific CD8⁺ T-cells in paired spleen and LN samples with tetramers collated (n=26 donors; spleen and n=24 donors; LN) or separate (n=1-6 donors). Donors which had 0 tetramer⁺ cells counted are represented as "x", and were included in statistics as 0. Open symbols represent <10 tetramer⁺ cells counted. **(b)** Proportion of IBV-specific CD8⁺ T_{rm} in paired spleen and LN with tetramers collated (n=11 donors each) or separate (n=1-3 donors). Statistical significance was determined using a two tailed Wilcoxon matched-pairs signed rank test. Source data are provided as a Source Data file.

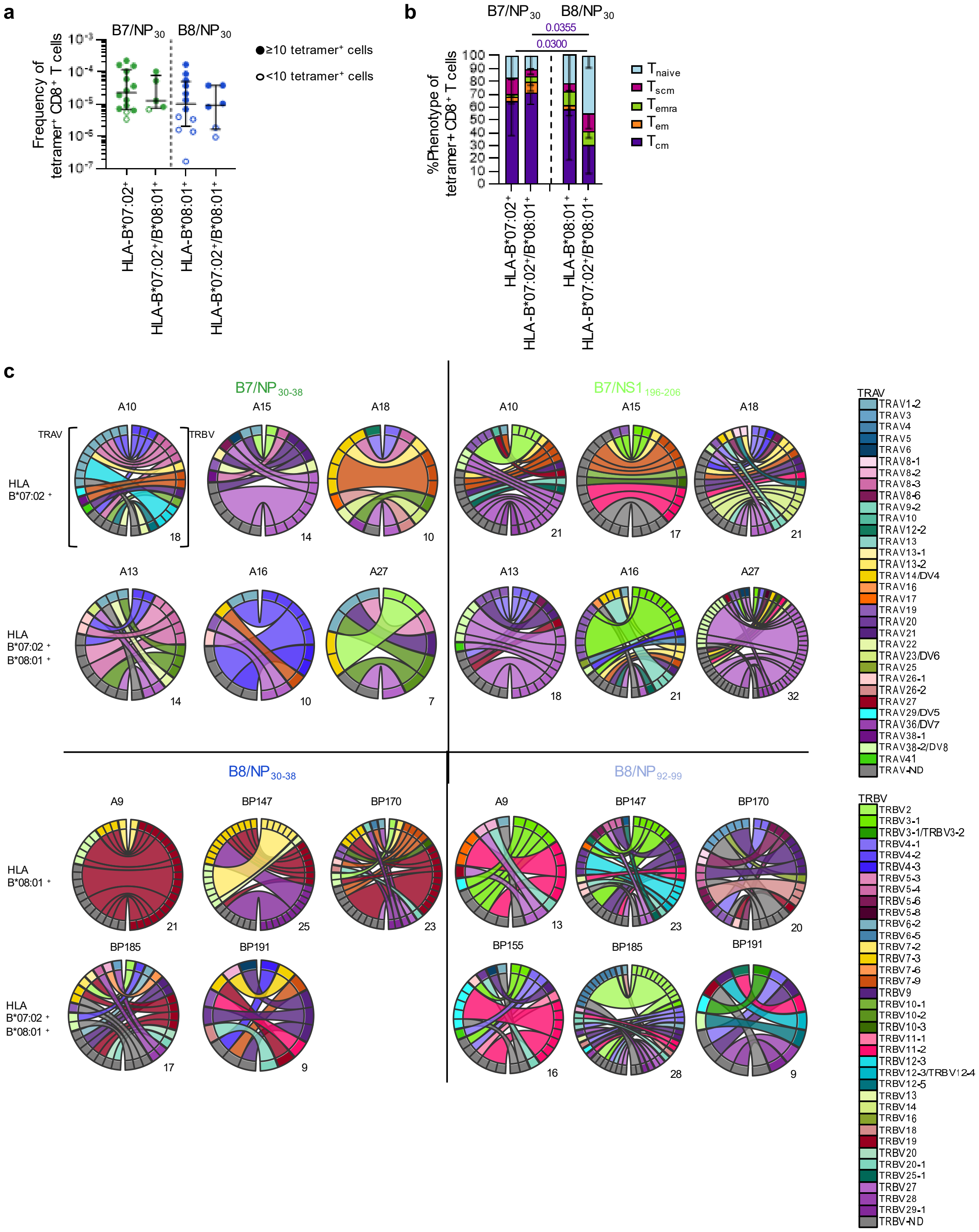

Supplementary Fig. 5. Analysis of TCR repertoire. (a) Frequency of B7/NP$_{30}$$^{+}$CD8^{+} and B8/NP$_{30}$$^{+}$CD8^{+} T-cells across HLA-B*07:02^{+} (n=14 donors), HLA-B*08:01^{+} (n=12) and HLA-B*07:02^{+}/B*08:01^{+} donors (n=7 donors). Open symbols represent <10 tetramer^{+} cells counted, excluded from phenotypic analysis. Statistical significance was determined with Mann-Whitney U test for frequency. Bars represent median and IQR. **(b)** Phenotype of B7/NP$_{30}$$^{+}$CD8^{+} and B8/NP$_{30}$$^{+}$CD8^{+} T-cells across HLA-B*07:02^{+} (n=12 donors), HLA-B*08:01^{+} (n=6) and HLA-B*07:02^{+}/B*08:01^{+} donors (n=7 donors). Statistical significance was determined by two-way ANOVA with Sidak's

test for multiple comparisons for phenotype. Bars represent mean and SD **(c)** Circos plots of TRAV and TRBV gene usage per donor. Left arch segment colours indicate TRAV usage, right outer arch colours depict TRBV usage. Connecting lines indicated TRAV-TRBV gene pairing and are coloured based on their TRBV usage and segmented based on their CRD3α and CDR3β sequence, the thickness is proportional to the number of TCR clones with the respective pair. The number of sequences considered for each circos plot is shown at the right bottom. Source data are provided as a Source Data file.

a) C1R.B*07:02

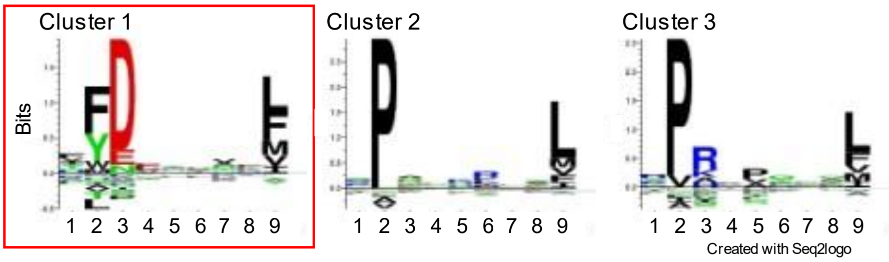

Cluster	Size
1 (HLA-C*04:01)	2507
2	6959
3	7169
Outliers	569

b) C1R.B*08:01

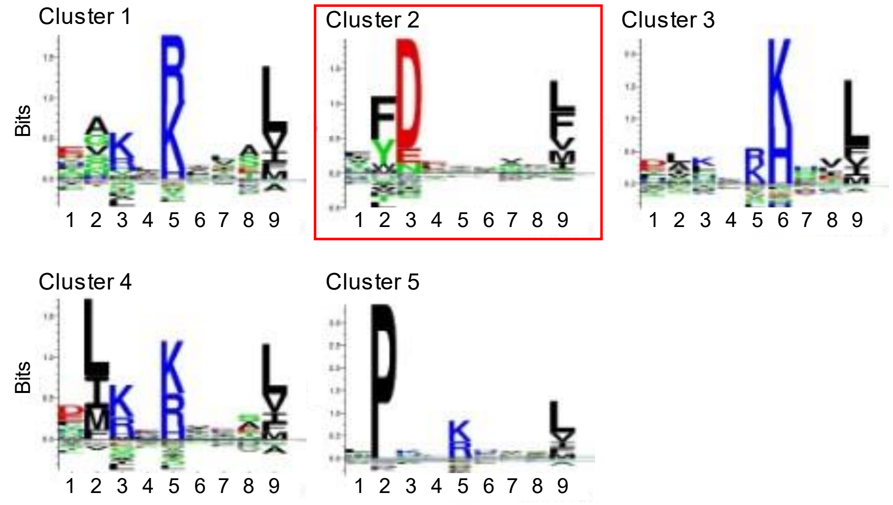

Cluster	Size
1	6318
2 (HLA-C*04:01)	2188
3	4845
4	7039
5	4488
Outliers	483

c) C1R.B*35:01

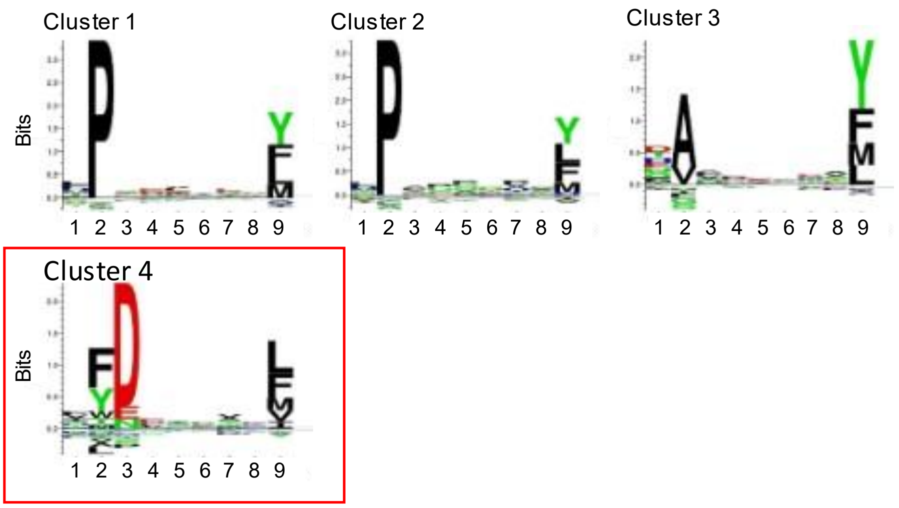

Cluster	Size
1	5947
2	5663
3	4139
4 (HLA-C*04:01)	3399
Outliers	478

Supplementary Fig. 6. Gibbs cluster analysis of IBV peptides. Peptide motifs for peptide sequence clusters generated via Gibbs cluster analysis of unfiltered 8-13 amino acid **(a)** C1R.B*07:02, **(b)** C1R.B*08:01 and **(c)** C1R.B*35:01 human peptide sequences identified at 1% FDR. Peptides from Cluster 1, aligning with the binding motif for HLA-C*04:01, were filtered from motif analyses for C1R-B*07:02, while Cluster 2 peptides were filtered from motif analyses for C1R.B*08:01 and Cluster 4 peptides were filtered from motif analyses for C1R.B*35:01.

Supplementary Table 1. Selected HLA-B*07:02, -B*08:01 and B*35:01 restricted IBV peptides.

HLA	Pool	Peptide Name	Sequence	Length
HLA-B*07:02	A	PB1+2$_{80-89}$	YPKIMSRVPM	10
		HA+3$_{211-220}$	RPNYRLGVPL	10
		PB1$_{706-714}$	KPVGQHSML	9
		HA$_{224-232}$	KPQKFTSSA	9
		NS1$_{121-129}$	YPSTPGRCL	9
		HA$_{255-263}$	LPQSGRIVV	9
		NP$_{472-481}$	RPIALSKQAV	10
		HA$_{91-99}$	IPSARVSIL	9
		NP$_{233-244}$	LPRRSGATGVAI	12
		HA$_{27-35}$	SPHVVKTAT	9
		NP+2$_{180-188}$	MIKPSTSAL	9
		NP$_{30-38}$	RPIIRPATL	9
		NS1$_{66-74}$	EPESKRMSL	9
	B	HA+3$_{82-92}$	MPQLHRSGRGL	11
		PB1$_{393-404}$	KPFFNEEGTASL	12
		NS1$_{253-265}$	KPIRAAETAVGVL	13
		NS1$_{196-206}$	HPNGYKSLSTL	11
		NA$_{458-466}$	TVTGVNMAL	9
		NP$_{536-546}$	IPIKQTIPNFF	11
		NS2$_{62-70}$	TIRLVTEEL	9
		HA$_{110-119}$	FPIMHDRTKI	10
HLA-B*08:01	C	PA$_{453-460}$	TVMMKYVL	8
		NS1$_{66-74}$	EPESKRMSL	9
		NS2$_{11-19}$	EWRMKKMAI	9
		NA$_{201-209}$	LLKIKYGEA	9
		NP$_{30-38}$	RPIIRPATL	9
		PB1$_{186-193}$	NIKKKLPA	8
		NP$_{407-414}$	EFKPRSAL	8
		NP$_{500-508}$	LLKMMNDSM	9
		NP$_{468-476}$	FAVERPIAL	9
		NP$_{173-181}$	FLKEEVKTM	9
		PB2$_{560-567}$	TLKAQFLL	8
		PB2$_{207-215}$	YMLERELVA	9
		NA$_{150-159}$	RNKLRHLISV	10
		NA$_{130-137}$	FALTHYAA	8
		NP$_{479-487}$	QAVRRMLSM	9
		NP$_{92-99}$	QMMVKAGL	8
		PA$_{458-465}$	YVLFHTSL	8
		PB2$_{187-195}$	LIKEKREKL	9
		NP$_{517-525}$	FIGKKMFQI	9
	D	HA$_{481-488}$	ERKLKKML	8
		NA$_{150-157}$	RNKLRHLI	8
		PA$_{132-139}$	FWKKKEKL	8
		HA$_{110-119}$	FPIMHDRTKI	10
		PB1$_{216-224}$	EYIKRALSL	9
		NS1$_{168-175}$	EGKFRLTI	8
		NS2$_{115-122}$	EVYSRQCL	8
		PB2$_{207-214}$	YMLERELV	8
		M1$_{170-177}$	VPGVRREM	8
		NP$_{74-82}$	EIKKSVYNM	9
		NA$_{414-422}$	EIKDKKCDV	9
		NP$_{396-403}$	DLRVLSAL	8
		PB2$_{594-601}$	SGFARAVL	8
		PB2$_{497-505}$	VSITKNLSL	9
		PA$_{131-139}$	YFWKKKEKL	9
		NP$_{263-271}$	ADRGLLRDI	9
		NP$_{377-385}$	DAKDKSQLF	9

HLA-B*35:01	E	PB1$_{22-30}$	FPYTGVPPY	9
		PB2$_{66-74}$	MANRIPLEY	9
		NP$_{468-476}$	FAVERPIAL	9
		NA$_{386-394}$	DPWADSDAL	9
		NS2$_{47-56}$	YPNLVKSTDY	10
		PB2$_{431-439}$	SPMYQLQRY	9
		M1$_{132-140}$	YLNPGNYSM	9
		NA$_{375-383}$	MGMGLYVKY	9
		NP$_{82-90}$	MVVKLGEFY	9
		NP$_{157-165}$	MVRDDKTIY	9
		NP$_{536-545}$	IPIKQTIPNF	10
		PA$_{399-407}$	VAAWVQTEM	9
		HA$_{231-239}$	SANGVTTHY	9
		NA$_{164-172}$	IPTVENSIF	9
		HA$_{336-344}$	CPIWVKTPL	9
		NP$_{536-544}$	IPIKQTIPN	9
		NA$_{40-50}$	FPSTEITAPTM	11
	F	NS1$_{260-268}$	TAVGVLSQF	9
		HA$_{110-118}$	**FPIMHDRTK**	9
		NA$_{386-396}$	DPWADSDALAF	11
		NA$_{111-119}$	APLIIREPF	9
		NP$_{71-80}$	TPTEIKKSVY	10
		NP$_{536-546}$	IPIKQTIPNFF	11
		NP$_{165-173}$	YFSPIRVTF	9
		PB2$_{277-285}$	NPLELAVEI	9
		HA$_{255-265}$	LPQSGRIVVDY	11
		HA$_{255-263}$	LPQSGRIVV	9
		HA$_{91-99}$	IPSARVSIL	9
		PB2$_{156-166}$	MPPDEASNVIM	11
		HA$_{307-315}$	EADCLHEKY	9
		PA$_{216-229}$	VPAGFSNFEGMRSY	14
		HA$_{163-171}$	NGNGFFATM	9
		PB1$_{486-494}$	TGMFEFTSM	9
		NP$_{157-166}$	MVRDDKTIYF	10
		HA$_{246-255}$	FPNQTEDGGL	10
		PB1$_{604-612}$	HIPEIVLKY	9
		HA$_{556-564}$	AASSLAVTL	9
		NA$_{458-466}$	TVTGVNMAL	9
	G	HA$_{110-122}$	**FPIMHDRTKIRQL**	13
		HA$_{255-266}$	LPQSGRIVVDYM	12
		PA$_{450-458}$	KASTVMMKY	9
		NA$_{440-448}$	SAATAIYCL	9
		PB1$_{57-65}$	ISDVTGCTM	9
		HA$_{110-119}$	FPIMHDRTKI	10
		HA+2$_{172-182}$	LPMETDFSQQW	11
		NA$_{2-12}$	LPSTIQTLTLF	11
		NA$_{386-395}$	DPWADSDALA	10
		NP$_{253-262}$	EAIRFIGRAM	10
		NP$_{30-38}$	RPIIRPATL	9
		NA$_{387-394}$	PWADSDAL	8
		HA$_{392-400}$	HGVAVAADL	9
		NA$_{56-64}$	NASNVQAVN	9
		PA$_{263-270}$	RPIGPHIY	8
		NP$_{173-181}$	FLKEEVKTM	9
		PB1$_{22-38}$	FPYTGVPPYSHGTGTGY	17

Bold indicates overlapping between peptides. Underline indicates immunogenic peptides.

Supplementary Table 2. Study donor demographics.

Cohort	ID	Age group	Tissue	HLA-A	HLA-A	HLA-B	HLA-B	HLA-C	HLA-C	Peptide screen	HLA confirmation	Tetramer testing	TAME	*Ex vivo* TCR	Tetramer staining
Adult	A1	Adult	PBMC	01:01	03:01	35:01	57:01						Yes		
Median 39 yrs	A2	Adult	PBMC	02:01	03:01	35:01	57:01	04:01	06:02				Yes		
Range 25-59 yrs	A3	Adult	PBMC	02:01	24:02	27:05	35:01	01:02	04:01				Yes		
42% F	A4	Adult	PBMC	24:02		07:02		07:02		Yes	Yes	Yes	Yes		
2 Unk sex	A5	Adult	PBMC	01:01	24:02	35:01	57:01	04:01	06:02	Yes	Yes	Yes	Yes		
	A6	Adult	PBMC	01:01	31:01	07:02	08:01	07:01	07:02	Yes	Yes	Yes	Yes		
	A7	Adult	PBMC	01:01	32:01	08:01	40:01	03:04	07:01	Yes	Yes	Yes	Yes		
	A8	Adult	PBMC	01:01	03:01	18:01	35:01	04:01	07:01	Yes	Yes	Yes	Yes		
	A9	Adult	PBMC	26:01	32:01	08:01	35:03	04:01	07:02		Yes		Yes	Yes	
	A10	Adult	PBMC	01:01	24:02	07:02	57:01	06:02	07:02				Yes	Yes	
	A11	Adult	PBMC	01:01	30:02	08:01	51:01	07:18	14:02	Yes	Yes	Yes	Yes	Yes	
	A12	Adult	PBMC	26:01	30:01	35:01	50:01	04:01	06:02	Yes	Yes	Yes	Yes		
	A13	Adult	PBMC	01:01	03:01	07:02	08:01	07:01	07:02	Yes	Yes	Yes	Yes	Yes	
	A14	Adult	PBMC	01:01	23:01	08:01	15:01	03:03	07:01	Yes	Yes	Yes	Yes	Yes	
	A15	Adult	PBMC	03:01	24:02	07:02	35:08	04:01	07:02	Yes	Yes	Yes	Yes	Yes	
	A16	Adult	PBMC	01:01	03:01	07:02	08:01	07:01	07:02	Yes	Yes	Yes	Yes	Yes	
	A17	Adult	PBMC	02:01	24:02	35:01	44:02	04:01	05:01	Yes	Yes	Yes	Yes		
	A18	Adult	PBMC	03:01	24:02	07:02	40:02	02:02	07:02	Yes	Yes	Yes	Yes	Yes	
	A19	Adult	PBMC	02:01	11:01	35:01	40:01	03:04	04:01	Yes	Yes	Yes	Yes		
	A20	Adult	PBMC	01:01		07:02	08:01	07:01	07:02	Yes	Yes	Yes	Yes	Yes	
	A21	Adult	PBMC	02:01	11:01	18:01	35:01	04:01	07:01	Yes	Yes	Yes	Yes		
	A22	Adult	PBMC	01:01	29:02	08:01	44:03	07:01	16:01		Yes		Yes		
	A23	Adult	PBMC	01:01	23:01	08:01	45:01	06:02	07:01		Yes		Yes		
	A24	Adult	PBMC	03:01	26:01	07:02	15:01				Yes		Yes		
	A25	Adult	PBMC	03:01	31:01	07:02		03:04	07:02		Yes		Yes		
	A26	Adult	PBMC	01:01	02:01	07:02	44:03	07:02	16:01		Yes		Yes		
	A27	Adult	PBMC	01:01	03:01	07:02	08:01	07:01	07:02		Yes		Yes	Yes	
	A28	Adult	PBMC	01:01		07:02	08:01	07:01	07:02				Yes		
Elderly	E1	Elderly	PBMC	02:01	32:01	07:02	44:03						Yes		
Median 73 yrs	E2	Elderly	PBMC	01:01		07:02	08:01	07:01	07:02				Yes		
Range 65-87 yrs	E3	Elderly	PBMC	02:01	03:01	07:02	44:03	07:02	16:01				Yes		
57% F	E4	Elderly	PBMC	01:01	03:01	08:01	35:01						Yes		
2 Unk sex	E5	Elderly	PBMC	01:01	02:01	07:02	15:01	03:04	07:02				Yes		
	E6	Elderly	PBMC	02:01	25:01	07:02	44:02	05:01	07:02				Yes		
	E7	Elderly	PBMC	01:01	24:02	08:01	57:01	07:01					Yes		
	E8	Elderly	PBMC	01:01	29:02	08:01	44:03	07:01	16:01				Yes		
	E9	Elderly	PBMC	01:01	03:01	08:01	40:02	02:02	07:01				Yes		
Child	C1	Child	PBMC	01:01	25:01	08:01	18:01	07:01	07:01				Yes		
Median 9 yrs	C2	Child	PBMC	24:02	33:01	07:02	15:01	03:03	07:02				Yes		
Range 1-16 yrs	C3	Child	PBMC	03:01		07:02	44:03	07:02	16:01				Yes		
50% F	C4	Child	PBMC	01:01	32:01	08:01	14:01	07:01	08:02				Yes		
	C5	Child	PBMC	03:01	30:02	07:02	18:01	05:01	07:02				Yes		
	C6	Child	PBMC	11:01	31:01	07:02	35:01	04:01	07:02				Yes		
Spleen	S1	Adult	Spleen	02:01	11:01	15:01	35:01	1	4				Yes		
Median 51 yrs	S2	Adult	Spleen	03:01	11:01	08:01	38:01	7	12				Yes		

Range 35-64 yrs	S3	Adult	Spleen	02:01	24:02	27:05	35:01	2	4	Yes	
27% F	S4	Adult	Spleen	02:01	24:02	27:05	35:01	2	4	Yes	
	S5	Adult	Spleen	01:01	31:01	08:01		7		Yes	
	S6	Adult	Spleen	1	2	8	13	6	7	Yes	
	S7	Adult	Spleen	1	68	7	18	7		Yes	
	S8	Adult	Spleen	2	3	7	57	6	7	Yes	
	S9	Adult	Spleen	24	31	7	14:02	7	8	Yes	
	S10	Adult	Spleen	2	25	7	8	7		Yes	
	S11	Adult	Spleen	01:01	24:02	08:01	13:01	03:04	07:01	Yes	
	S12	Adult	Spleen	01:01		08:01		07:01		Yes	
	S13	Adult	Spleen	2	11	7	44	05	07	Yes	
	S14	Elderly	Spleen	3	24	35	39	04	07	Yes	
	S15	Adult	Spleen	03	25	07		07		Yes	
Lymph nodes	N1	Adult	Lymph node	03:01	11:01	08:01	38:01	7	12	Yes	
Median 49 yrs	N2	Adult	Lymph node	01:01	31:01	08:01		7		Yes	
Range 35-64 yrs	N3	Adult	Lymph node	1	2	8	13	6	7	Yes	
27% F	N4	Adult	Lymph node	1	68	7	18	7		Yes	
	N5	Adult	Lymph node	2	3	7	57	6	7	Yes	
	N6	Adult	Lymph node	24	31	7	14:02	7	8	Yes	
	N7	Adult	Lymph node	2	25	7	8	7		Yes	
	N8	Adult	Lymph node	01:01		08:01		07:01		Yes	
	N9	Adult	Lymph node	2	11	7	44	05	07	Yes	
	N10	Elderly	Lymph node	3	24	35	39	04	07	Yes	
	N11	Adult	Lymph node	03	25	07		07		Yes	
Tonsil	T1	Child	Tonsil	01:01	25:01	08:01	18:01	07:01	07:01	Yes	
Median 10 yrs	T2	Child	Tonsil	01:01	25:01	07:02	08:01	07:01	07:02	Yes	
Range 6-34 yrs	T3	Child	Tonsil	03:01	32:01	07:02	08:01	07:01	07:02	Yes	
53% F	T4	Child	Tonsil	24:02	33:01	07:02	15:01	03:03	07:02	Yes	
	T5	Child	Tonsil	02:01		08:01	44:02	05:01	07:01	Yes	
	T6	Child	Tonsil	03:01		07:02	44:03	07:02	16:01	Yes	
	T7	Child	Tonsil	01:01	32:01	08:01	14:01	07:01	08:02	Yes	
	T7	Child	Tonsil	02:01	23:01	35:01	49:01	04:01	07:01	Yes	
	T8	Adult	Tonsil	01:01	24:02	07:02	40:01	03:04	07:02	Yes	
	T9	Child	Tonsil	03:01	30:02	07:02	18:01	05:01	07:02	Yes	
	T10	Child	Tonsil	11:01	31:01	07:02	35:01	04:01	07:02	Yes	
	T11	Adult	Tonsil	01:01	02:01	08:01	51:01	07:01	14:02	Yes	
	T12	Adult	Tonsil	01:01	25:01	07:02	18:01	07:02	12:03	Yes	
	T13	Child	Tonsil	01:01	03:01	07:02	08:01	07:01	07:02	Yes	
	T14	Adult	Tonsil	02:01	03:01	07:02	40:01	03:04	07:02	Yes	
Lung	L1	Adult	Lung	2	32	7	8	7	7		Yes
Median 26 yrs	L2	Adult	Lung	1	3	8	35	4	7		Yes
Range 22-41 yrs	L3	Adult	Lung	7	8	7	8	7			Yes
33% F	L4	Unknown	Lung	2	31	7	44	5	7		Yes
3 Unk age/sex	L5	Unknown	Lung	1	25	8	58	7	7		Yes
	L6	Unknown	Lung	1	3	35	55	3	4		Yes

F, female; Unk, unknown.

Supplementary Table 3. Immunogenic B7-I, B8-I and B35-I pools.

Pool H	Pool I	Pool J
$HA_{255-263}$	NP_{30-38}	$HA_{231-239}$
NP_{30-38}	NP_{92-99}	$NP_{165-173}$
$NP_{233-244}$	$NP_{468-477}$	$NP_{468-476}$
$NS1_{196-206}$	$NP_{479-487}$	$NS1_{260-268}$
$NS2_{62-70}$		$PB2_{66-70}$

Supplementary Table 4. Data collection and refinement statistics.

	**B*07:02/NS1$_{196-206}$ (8TUB)**	**B*07:02/NP$_{30-38}$ (8TUH)**
Wavelength	0.95366	0.95366
Resolution range	43.36 - 2.4 (2.486 - 2.4)	48.03 - 2.2 (2.27 - 2.2)
Space group	P 1	P 61 2 2
Unit cell (a, b, c)	49.2 90.7 104.6	57.2 57.2 396.4
(α, β, γ)	97.5 93.8 90.2	90 90 120
Total reflections	186122 (18249)	442482 (33657)
Unique reflections	66085 (6521)	21009 (1718)
Multiplicity	2.8 (2.8)	21.1 (19.6)
Completeness (%)	94.46 (93.12)	100 (100)
Mean I/sigma(I)	6.49 (1.10)	7.9 (2.4)
R-pim	0.13 (0.34)	0.064 (0.30)
CC1/2	0.953 (0.706)	0.993 (0.885)
R-work	0.197	0.178
R-free	0.237	0.241
Number of non-hydrogen atoms	13217	3320
macromolecules	12627	3203
ligands	77	28
solvent	559	89
RMS(bonds)	0.021	0.007
RMS(angles)	1.35	0.88
Ramachandran favoured (%)	97.36	97.7
Ramachandran allowed (%)	2.64	2.3
Ramachandran outliers (%)	0	0
Rotamer outliers (%)	0.45	2.6
B factor	37.77	43.3

Supplementary Table 5. Expanded clonotypes

B7/NP$_{30}$

Clone ID	TRAV	TRAJ	CDR3α	CDR3α length	TRBV	TRBJ	CDR3β	CDR3β length	BP146	BP155	BP184	BP185	BP189	KK102
A	TRAV1-2	TRAJ33	CAVNDGSNYQLIW	8	TRBV12-3	TRBJ2-1	CASSSPPSGGGYEQFF	11	3 (17%)					
Q					TRBV10-2	TRBJ1-5	CASSEETGNQPQHF	9		2 (14%)				
R	TRAV26-1	TRAJ42	CIVRLRTNYGGSQGNLIF	13	TRBV5-4	TRBJ1-3	CASSFDRGTGNTIYF	10		2 (14%)				
S					TRBV5-3	TRBJ1-3	CASSFDRGTGNTIYF	10		2 (14%)				
T					TRBV5-4	TRBJ1-3	CASSFDRGTGNTIYF	10		2 (14%)				
AA					TRBV27	TRBJ1-3	CASSPYGGPGNTIYF	10			5 (36%)			
AK					TRBV4-2	TRBJ1-1	CASSQSSGGGRSEAFF	11				3 (30%)		
AL	TRAV1-2	TRAJ28	CAVRDSGAGSYQLTF	10	TRBV4-2	TRBJ1-1	CASSQSSGGGRSEAFF	11				2 (20%)		
AM					TRBV4-3	TRBJ2-3	CASSQPSQGGRLEQYF	11				2 (20%)		
AQ	TRAV14/DV4	TRAJ44	CAMREVGGTASKLTF	10	TRBV7-9	TRBJ1-6	CASSIYPGQLYNSPLHF	12					3 (30%)	
AY	TRAV14/DV4	TRAJ18	CAITIEGSTLGRLYF	10	TRBV2	TRBJ1-4	CAGRGRPNEKLFF	8						2 (29%)
								Other singletons	15 (83%)	6 (43%)	9 (64%)	3 (30%)	7 (70%)	5 (71%)
								**Total**	**18**	**14**	**14**	**10**	**10**	**7**

B8/NP$_{30}$

Clone ID	TRAV	TRAJ	CDR3α	CDR3α length	TRBV	TRBJ	CDR3β	CDR3β length	BP140	BP147	BP170	BP185	BP191
A					TRBV19	TRBJ1-1	CASSMIPDMNTEAFF	10	5 (24%)		7 (30%)		
B	TRAV38-2/DV8	TRAJ42	CAPNYGGSQGNLIF	9	TRBV19	TRBJ2-2	CASGRIAGELFF	7	4 (19%)				
C					TRBV19	TRBJ1-1	CASSMVPDMNTEAFF	10	3 (14%)				
D					TRBV19	TRBJ2-7	CASSLVGGEQYF	7	2 (10%)				

L					TRBV29-1	TRBJ1-2	CSVVQGAECTF	6		6 (24%)				
M	TRAV38-2/DV8	TRAJ40	CAPSGTYKYIF	6	TRBV7-2	TRBJ2-7	CASSLVGGEQYF	7		6 (24%)				
N	TRAV14/DV4	TRAJ12	CAMSRQGMDSSYKLIF	11	TRBV29-1	TRBJ1-2	CSVVQGAECTF	6		5 (20%)				
O	TRAV14/DV4	TRAJ53	CAMRGGGSNYKLTF	9	TRBV19	TRBJ1-1	CASSSIPDFNTEAFF	10		2 (8%)				
V	TRAV14/DV4	TRAJ33	CAMREGGSNYQLIW	9	TRBV19	TRBJ1-1	CASSMIPDMNTEAFF	10			2 (9%)			
								Other singletons	7 (33%)	6 (24%)	14 (61%)	17 (100%)	9 (100%)	
								**Total**	**21**	**25**	**23**	**17**	**9**	

B7/NS1$_{196}$

Clone ID	TRAV	TRAJ	CDR3α	CDR3α length	TRBV	TRBJ	CDR3β	CDR3β length	KK102	BP146	BP155	BP184	BP185	BP189
A	TRAV38-2/DV8	TRAJ33	CAYRSAVGYQLIW	8	TRBV27	TRBJ1-5	CASSQYSNQPQHF	8	8 (25%)					
B					TRBV27	TRBJ1-5	CASSQYSNQPQHF	8	5 (16%)					
C					TRBV27	TRBJ1-1	CASSLMGNTEAFF	8	2 (6%)	1 (5%)				
D	TRAV38-2/DV8	TRAJ43	CAYRSAVTYDMRF	8	TRBV27	TRBJ1-5	CASSLYGNQPQHF	8	2 (6%)					
E					TRBV28	TRBJ1-1	CASSLSRGWTEAFF	9	2 (6%)					
S	TRAV19	TRAJ4	CALTRPGGGYNKLIF	10	TRBV2	TRBJ2-1	CASRGLQGRIDYNEQFF	12		4 (19%)				
T					TRBV27	TRBJ2-7	CASRIYAGSPYEQYF	10		2 (10%)				
AI					TRBV27	TRBJ2-1	CASSPHPGTSGGASYNEQFF	15			3 (17%)			
AJ	TRAV38-2/DV8	TRAJ30	CAYRSAIQDKIIF	8	TRBV27	TRBJ2-1	CASSPHPGTSGGASYNEQFF	15			2 (11%)			
AK	TRAV38-2/DV8	TRAJ41	CAYRSATGYALNF	8	TRBV27	TRBJ2-1	CASSLYGSVQNEQFF	10			2 (11%)			
AL	TRAV3	TRAJ27	CAVRDIGTNAGKSTF	10	TRBV27	TRBJ2-2	CASSYRGLSGELFF	9			2 (11%)			

AM	TRAV19	TRAJ13	CALSEYSGGYQKVTF	10	TRBV9	TRBJ2-1	CASSVATSGGSQQFF	10			2 (11%)			
AU					TRBV7-9	TRBJ1-1	CASNGQGDTEAFF	8				2 (12%)		
AV	TRAV3	TRAJ28	CAVRAPGAGSYQLTF	10								2 (12%)		
AW	TRAV3	TRAJ28	CAVRDPGAGSYQLTF	10								2 (12%)		
AX					TRBV11-2	TRBJ1-1	CASSPGSTEAFF	7				2 (12%)		
AY					TRBV11-2	TRBJ2-2	CASSLGVEELFF	7				2 (12%)		
BG	TRAV19	TRAJ4	CALLLFSGGYNKLIF	10	TRBV3-1	TRBJ2-5	CASSPREGGETQYF	9					7 (33%)	
BH	TRAV14/DV4	TRAJ10	CAMREGPELTGGGNKLTF	13	TRBV20-1	TRBJ1-2	CSAKRNRAYHYGYTF	10					3 (14%)	
BT					TRBV14	TRBJ2-7	CASATSGTYYEQYF	9						2 (10%)
			Other singletons						13 (41%)	14 (67%)	7 (39%)	7 (41%)	11 (52%)	19 (90%)
			**Total**						**32**	**21**	**18**	**17**	**21**	**21**

B8/NP_{92}

Clone ID	TRAV	TRAJ	CDR3α	CDR3α length	TRBV	TRBJ	CDR3β	CDR3β length	BP140	BP147	BP155	BP170	BP185	BP191
A	TRAV17	TRAJ31	CATDESNARLMF	7	TRBV11-2	TRBJ2-7	CASSLIRTWDEQYF	9	3 (23%)					
B					TRBV11-2	TRBJ2-7	CASSLIRTWDEQYF	9	2 (15%)					
K					TRBV3-1	TRBJ2-5	CASSQWGRPQETQYF	10		3 (13%)				
L	TRAV20	TRAJ45	CAVRTYSGGGADGLTF	11	TRBV12-3	TRBJ2-3	CASAPRRDRGTDTQYF	11		3 (13%)				
M					TRBV9	TRBJ2-3	CASSPSELAGGRLDTQYF	13		2 (9%)				
N	TRAV8-3	TRAJ20	CACGDYKLSF	5	TRBV9	TRBJ2-3	CASSPSELAGGRLDTQYF	13		2 (9%)				
O	TRAV8-6	TRAJ32	CAVTNGGATNKLIF	9	TRBV3-1	TRBJ2-5	CASSQWGRPQETQYF	10		2 (9%)				

AA	TRAV29/DV5	TRAJ49	CAANTGNQFYF	6	TRBV11-2	TRBJ2-2	CASSWRTGNSGELFF	10			4 (25%)			
AB					TRBV11-2	TRBJ2-2	CASSWRTGNSGELFF	10			3 (19%)			
AL	TRAV3	TRAJ5	CAVAMDTGRRALTF	9								3 (15%)		
AM	TRAV3	TRAJ5	CAVAMDTGRRALTF	9	TRBV9	TRBJ2-3	CASSNYPGGARTDTQYF	12				3 (15%)		
AN	TRAV8-6	TRAJ52	CAVSLNAGGTSYGKLTF	12	TRBV18	TRBJ2-7	CASSPPRNMEPSYEQYF	12				3 (15%)		
AO	TRAV19	TRAJ48	CALSEAPSNFGNEKLTF	12	TRBV4-1	TRBJ2-6	CASSHRDRGISGANVLTF	13				2 (10%)		
AP	TRAV8-1	TRAJ13	CAVNMALGGGYQKVTF	11	TRBV5-6	TRBJ2-7	CASSPPARDRDHEQYF	11				2 (10%)		
AQ	TRAV8-3	TRAJ20	CAVGDYKLSF	5	TRBV9	TRBJ2-3	CASSPSQTSGGRTDTQYF	13				2 (10%)		
AW	TRAV4	TRAJ39	CLVGDSGNAGNMLTF	10	TRBV2	TRBJ1-4	CASRMTGGKTNEKLFF	11					8 (29%)	
AX					TRBV2	TRBJ1-4	CASRMTGGKTNEKLFF	11					2 (7%)	
AY	TRAV4	TRAJ45	CLVMDSGGGADGLTF	10									2 (7%)	
			Other singletons						8 (62%)	11 (48%)	9 (56%)	5 (25%)	16 (57%)	9 (100%)
			**Total**						**13**	**23**	**16**	**20**	**28**	**9**

Supplementary Table 6. Clonotype counts of B7/NP$_{30-38}$.

Clone ID	TRAV	TRAJ	CDR3α	CDR3α length	TRBV	TRBJ	CDR3β	CDR3β length	BP146	BP155	BP184	BP185	BP189	KK102
A	TRAV1-2	TRAJ33	CAVNDGSNYQLIW	8	TRBV12-3	TRBJ2-1	CASSSPPSGGGYEQFF	11	3					
B	TRAV1-2	TRAJ36	CAVREGTGANNLFF	9	TRBV5-4	TRBJ1-5	CASSDRDRGGNQPQHF	11	1					
C	TRAV1-2	TRAJ36	CAVREGTGANNLFF	9	TRBV4-2	TRBJ2-7	CASSDREGGPHEQYF	10	1					
D	TRAV1-2	TRAJ36	CAVREGTGANNLFF	9	TRBV5-4	TRBJ2-7	CASSDREGGPHEQYF	10	1					
E	TRAV1-2	TRAJ36	CAVREGTGANNLFF	9	TRBV4-2	TRBJ1-2	CASSPREAGGISGYTF	11	1					
F	TRAV1-2	TRAJ36	CAVREGTGANNLFF	9	TRBV5-4	TRBJ2-2	CASSDRDVTGELFF	9	1					
G	TRAV1-2	TRAJ36	CAVREGTGANNLFF	9					1					
H					TRBV5-4	TRBJ2-2	CASSDRDVTGELFF	9	1					
I	TRAV29/DV5	TRAJ53	CAASGGSNYKLTF	8	TRBV7-9	TRBJ1-1	CASRQEGRPMNTEAFF	11	1					
J	TRAV38-1	TRAJ57	CAFLDQGGSEKLVF	9	TRBV7-9	TRBJ1-1	CASRQEGRPMNTEAFF	11	1					
K	TRAV1-2	TRAJ24	CAVRGDSWGKLQF	8	TRBV7-2	TRBJ1-1	CASRENRASNTEAFF	10	1					
L	TRAV41	TRAJ18	CAGFDRGSTLGRLYF	10	TRBV9	TRBJ2-1	CASSTPSSYNEQFF	9	1					
M					TRBV13	TRBJ2-6	CASSARSTGGFGANVLTF	13	1					
N					TRBV10-1	TRBJ2-7	CASSGRDRDLVEQYF	10	1					
O					TRBV4-2	TRBJ2-3	CASSPRGRGGREDTQYF	12	1					
P					TRBV12-5	TRBJ2-7	CASVQGGSGGRGSYEQYF	13	1					
Q					TRBV10-2	TRBJ1-5	CASSEETGNQPQHF	9		2				
R	TRAV26-1	TRAJ42	CIVRLRTNYGGSQGNLIF	13	TRBV5-4	TRBJ1-3	CASSFDRGTGNTIYF	10		2				
S					TRBV5-3	TRBJ1-3	CASSFDRGTGNTIYF	10		2				
T					TRBV5-4	TRBJ1-3	CASSFDRGTGNTIYF	10		2				
U	TRAV1-2	TRAJ9	CAVRDGGGFKTIF	8	TRBV10-2	TRBJ1-5	CASSEETGNQPQHF	9		1				
V	TRAV1-2	TRAJ38	CAVNAGNNRKLIW	8	TRBV13	TRBJ2-7	CASSLAGTGGRFSYEQYF	13		1				
W	TRAV1-2	TRAJ38	CAVNAGNNRKLIW	8						1				
X	TRAV23/DV6	TRAJ43	CAATFLRDMRF	6	TRBV27	TRBJ2-2	CASSYYRLAGELFF	9		1				
Y					TRBV4-2	TRBJ2-3	CASRIWDGGASTDTQYF	12		1				
Z					TRBV27	TRBJ1-2	CASSLWKGAGYTF	8		1				
AA					TRBV27	TRBJ1-3	CASSPYGGPGNTIYF	10			5			
AB	TRAV1-2	TRAJ32	CAVNDGATNKLIF	8	TRBV2	TRBJ2-6	CARAFESGANVLTF	9			1			
AC	TRAV1-2	TRAJ43	CAVRDGGRNDMRF	8	TRBV9	TRBJ2-7	CASSVEAGPNEQYF	9			1			
AD					TRBV9	TRBJ2-7	CASSVEAGPNEQYF	9			1			
AE	TRAV8-6	TRAJ17	CAVSEISAAGNKLTF	10	TRBV27	TRBJ1-3	CASSPYGGPGNTIYF	10			1			
AF	TRAV13-1	TRAJ45	CAALEAGGGADGLTF	10	TRBV9	TRBJ2-3	CASSVFGVQRGFGTDTQYF	14			1			
AG	TRAV19	TRAJ53	CALPDGGSNYKLTF	9	TRBV13	TRBJ2-7	CASSSRGSGGASSYEQYF	13			1			
AH	TRAV38-1	TRAJ56	CAFMKLWGGANSKLTF	11	TRBV5-4	TRBJ2-5	CASSLIGEETQYF	8			1			
AI	TRAV6	TRAJ9	CALELLTGNTGGFKTIF	12	TRBV27	TRBJ1-5	CASSLWQGGQPQHF	9			1			
AJ					TRBV27	TRBJ2-3	CASSSLPGGGVDTQYF	11			1			
AK					TRBV4-2	TRBJ1-1	CASSQSSGGGRSEAFF	11				3		
AL	TRAV1-2	TRAJ28	CAVRDSGAGSYQLTF	10	TRBV4-2	TRBJ1-1	CASSQSSGGGRSEAFF	11				2		
AM					TRBV4-3	TRBJ2-3	CASSQPSQGGRLEQYF	11				2		
AN	TRAV14/DV4	TRAJ24	CAMREGRTTDSWGKLQF	12	TRBV7-9	TRBJ2-5	CASSLGREETQYF	8				1		
AO	TRAV19	TRAJ44	CALSEAISTGTASKLTF	12	TRBV27	TRBJ2-2	CASSTWSQNTGELFF	10				1		
AP	TRAV26-1	TRAJ44	CIAFYYAALPVRLTF	10	TRBV27	TRBJ1-5	CASSTGQGEGLYSNQPQHF	14				1		
AQ	TRAV14/DV4	TRAJ44	CAMREVGGTASKLTF	10	TRBV7-9	TRBJ1-6	CASSIYPGQLYNSPLHF	12					3	
AR	TRAV12-2	TRAJ45	CAVSDSGGGADGLTF	10	TRBV4-1	TRBJ2-3	CASSQVVAFGDTQYF	10					1	
AS	TRAV14/DV4	TRAJ52	CAMREGGGTSYGKLTF	11	TRBV7-2	TRBJ2-7	CASSLAGTSGYTYEQYF	12					1	
AT	TRAV14/DV4	TRAJ17	CAMSLEGGAGNKLTF	10	TRBV5-4	TRBJ2-1	CASSLRQALRNEQFF	10					1	
AU	TRAV17	TRAJ53	CATDASKSGGSNYKLTF	12	TRBV18	TRBJ1-1	CASSPGEGMLVSEAFF	11					1	
AV	TRAV22	TRAJ28	CAVDVDSGAGSYQLTF	11	TRBV14	TRBJ1-1	CASSDNRVWTEAFF	9					1	
AW	TRAV36/DV7	TRAJ37	CAVFSSNTGKLIF	8	TRBV10-2	TRBJ2-1	CASSGVGSGRPNSYNEQFF	14					1	
AX					TRBV27	TRBJ1-5	CASSQWGGQPQHF	8					1	
AY	TRAV14/DV4	TRAJ18	CAITIEGSTLGRLYF	10	TRBV2	TRBJ1-4	CAGRGRPNEKLFF	8						2
AZ	TRAV1-2	TRAJ36	CAVREGTGANNLFF	9	TRBV5-3	TRBJ1-1	CASSLREMGGKAEAFF	11						1
BA	TRAV1-2	TRAJ15	CAVRDGAGTALIF	8	TRBV9	TRBJ2-5	CASSVEGGGAQETQYF	11						1
BB	TRAV1-2	TRAJ38	CAVNAGNNRKLIW	8	TRBV10-2	TRBJ2-7	CASILGTSGREPYEQYF	12						1
BC					TRBV10-2	TRBJ2-7	CASILGTSGREPYEQYF	12						1
BD					TRBV27	TRBJ1-4	CASSLYRGIGLFF	8						1
								**Grand total**	**18**	**14**	**14**	**10**	**10**	**7**

Red denotes truncated CDR3 regions.

Supplementary Table 7. Clonotype counts of B8/NP$_{30-38}$.

Clone ID	TRAV	TRAJ	CDR3α	CDR3α length	TRBV	TRBJ	CDR3β	CDR3β length	BP140	BP147	BP170	BP185	BP191
A					TRBV19	TRBJ1-1	CASSMIPDMNTEAFF	10	5		7		
B	TRAV38-2/DV8	TRAJ42	CAPNYGGSQGNLIF	9	TRBV19	TRBJ2-2	CASGRIAGELFF	7	4				
C					TRBV19	TRBJ1-1	CASSMVPDMNTEAFF	10	3				
D					TRBV19	TRBJ2-7	CASSLVGGEQYF	7	2				
E	TRAV14/DV4	TRAJ53	CAMRGGGSNYKLTF	9	TRBV19	TRBJ1-1	CASSMIPDMNTEAFF	10	1				
F					TRBV19	TRBJ2-2	CASGRIAGELFF	7	1				
G	TRAV14/DV4	TRAJ28	CAMREGDTSNSGAGSYQLTF	15	TRBV19	TRBJ1-1	CASSMVPDMNTEAFF	10	1				
H					TRBV19	TRBJ1-1	CASSVIPDMNTEAFF	10	1				
I	TRAV13-2	TRAJ13	CAEISGGYQKVTF	8	TRBV7-2	TRBJ1-1	CASSLVGATAFF	7	1				
J	TRAV14/DV4	TRAJ16	CAMREGGQKLLF	7	TRBV19	TRBJ1-6	CATSTVPDYNSPLHF	10	1				
K	TRAV38-2/DV8	TRAJ53	CAPYSGGSNYKLTF	9	TRBV19	TRBJ2-1	CASSLIGNEQFF	7	1				
L					TRBV29-1	TRBJ1-2	CSVVQGAECTF	6		6			
M	TRAV38-2/DV8	TRAJ40	CAPSGTYKYIF	6	TRBV7-2	TRBJ2-7	CASSLVGGEQYF	7		6			
N	TRAV14/DV4	TRAJ12	CAMSRQGMDSSYKLIF	11	TRBV29-1	TRBJ1-2	CSVVQGAECTF	6		5			
O	TRAV14/DV4	TRAJ53	CAMRGGGSNYKLTF	9	TRBV19	TRBJ1-1	CASSSIPDFNTEAFF	10		2			
P	TRAV14/DV4	TRAJ53	CAMREDSGGSNYKLTF	11	TRBV19	TRBJ1-1	CASSMIPDMNTEAFF	10		1			
Q	TRAV14/DV4	TRAJ53	CAMRGGSGGSNYKLTF	11	TRBV19	TRBJ1-1	CASSIIPDMNTEAFF	10		1			
R	TRAV14/DV4	TRAJ6	CAMRESASGGSYIPTF	11	TRBV19	TRBJ1-1	CASSIIPDMNTEAFF	10		1			
S					TRBV19	TRBJ1-1	CASSNIPDMNTEAFF	10		1			
T	TRAV14/DV4	TRAJ53	CAMRDSGGSNYKLTF	10	TRBV19	TRBJ1-1	CASSNVPDMNTEAFF	10		1			
U	TRAV38-2/DV8	TRAJ43	CAYRAPYNNNDMRF	9	TRBV13	TRBJ1-2	CASSQARDRGNGYTF	10		1			
V	TRAV14/DV4	TRAJ33	CAMREGGSNYQLIW	9	TRBV19	TRBJ1-1	CASSMIPDMNTEAFF	10			2		
W	TRAV14/DV4	TRAJ53	CAMREGGGGSNYKLTF	11	TRBV19	TRBJ1-1	CASSIIPDMNTEAFF	10			1		
X					TRBV27	TRBJ2-1	CASSPSGTGANEQFF	10			1		
Y					TRBV7-9	TRBJ1-2	CASSLEGGQHGYTF	9			1		
Z					TRBV7-9	TRBJ2-1	CASSSSGSSYNEQFF	10			1		
AA	TRAV1-2	TRAJ33	CAVRDDSNYQLIW	8	TRBV28	TRBJ1-5	CASTAQGFSNQPQHF	10			1		
AB	TRAV14/DV4	TRAJ5	CAMSLHDTGRRALTF	10	TRBV7-2	TRBJ1-5	CASSLNFVGLNQPQHF	11			1		
AC	TRAV19	TRAJ34	CALSEAYNTDKLIF	9	TRBV10-3	TRBJ1-1	CAIANRGTEAFF	7			1		
AD	TRAV19	TRAJ5	CALSDPYGGRRALTF	10	TRBV29-1	TRBJ2-7	CSAVGLAGVRSYEQYF	11			1		
AE	TRAV26-1	TRAJ48	CIVRRQSNFGNEKLTF	11	TRBV7-9	TRBJ1-6	CASSLAREYSPLHF	9			1		
AF	TRAV26-2	TRAJ34	CILRDAYNTDKLIF	9	TRBV20-1	TRBJ1-1	CSARRSDRVNTEAFF	10			1		
AG	TRAV38-2/DV8	TRAJ12	CAYRSAPDSSYKLIF	10	TRBV2	TRBJ2-2	CASRGLSNTGELFF	9			1		
AH	TRAV38-2/DV8	TRAJ33	CAYRSAMDSNYQLIW	10	TRBV19	TRBJ2-2	CASSTIGPYGELFF	9			1		
AI	TRAV4	TRAJ30	CLLRDDKIIF	5	TRBV7-9	TRBJ1-1	CASSPHQEVTEAFF	9			1		
AJ	TRAV9-2	TRAJ43	CALSEIDMRF	5	TRBV29-1	TRBJ1-1	CSAGTGSANTEAFF	9			1		
AK					TRBV19	TRBJ1-1	CASSMVPDMNTEAFF	10				1	
AL					TRBV19	TRBJ2-1	CASNPSAGTLYNEQFF	11				1	
AM					TRBV20-1	TRBJ2-2	CSARPRRSAGELFF	9				1	
AN					TRBV20	TRBJ2-2	CSAEGLAGGKNTGELFF	12				1	
AO					TRBV6-2	TRBJ1-6	CASSYSKMTAYNSPLHF	12				1	
AP					TRBV9	TRBJ1-6	CASSVGGISPLHF	8				1	
AQ					TRBV9	TRBJ2-7	CASSPGQGVYEQYF	9				1	
AR	TRAV13-1	TRAJ41	CAASQRMNSGYALNF	10	TRBV19	TRBJ1-5	CASSLGTLQPQHF	8				1	
AS	TRAV13-2	TRAJ26	CAEKSYYGQNFVF	8	TRBV2	TRBJ1-1	CASSGRTGNTEAFF	9				1	
AT	TRAV14/DV4	TRAJ23	CAMREFYNQGGKLIF	10	TRBV19	TRBJ2-2	CASSVSRDRNGELFF	10				1	
AU	TRAV14/DV4	TRAJ26	CAMRGGYGQNFVF	8								1	
AV	TRAV19	TRAJ15	CALSEATRDQAGTALIF	12								1	
AW	TRAV3	TRAJ31	CAVRNARLMF	5	TRBV28	TRBJ2-3	CASSQGQGIADTQYF	10				1	
AX	TRAV38-2/DV8	TRAJ39	CAYRSSVYNNAGNMLTF	12	TRBV4-2	TRBJ2-1	CASSQNSVGSYNEQFF	11				1	
AY	TRAV38-2/DV8	TRAJ56	CAYPTGANSKLTF	8								1	
AZ	TRAV8-2	TRAJ27	CAVRVNTNAGKSTF	9	TRBV7-6	TRBJ2-7	CASSSSYEQYF	6				1	
BA	TRAV8-3	TRAJ29	CAVAPLNSGNTPLVF	10	TRBV28	TRBJ2-3	CASSFARTRWEQYF	9				1	
BB					TRBV4-3	TRBJ1-6	CASSQARDPQGILHF	10					1
BC					TRBV7-9	TRBJ2-1	CASSSAGVEQFF	7					1
BD					TRBV9	TRBJ1-2	CASSPGGVYGYTF	8					1
BE	TRAV14/DV4	TRAJ48	CAIWTFGGEKLTF	8	TRBV19	TRBJ2-3	CASSRSLTDTQYF	8					1
BF	TRAV19	TRAJ22	CALTPSSGSARQLTF	10	TRBV11-2	TRBJ1-2	CASSFFGAQQVDGYTF	11					1
BG	TRAV20	TRAJ34	CAAKGYNTDKLIF	8	TRBV9	TRBJ2-2	CASKRNTGELFF	7					1
BH	TRAV26-2	TRAJ12	CILRDRDSSYKLIF	9	TRBV20-1	TRBJ2-5	CSARDPAPEETQYF	9					1
BI	TRAV6	TRAJ42	CALENYGGSQGNLIF	10	TRBV9	TRBJ2-7	CASSASAEFSSYEQYF	11					1

BJ	TRAV8-2	TRAJ42	CVVSEEGIIRIGSQGNLIF	14	TRBV7-3	TRBJ2-1	CASSPTSGSVYEQFF	10					1
								**Grand total**	**21**	**25**	**23**	**17**	**9**

Red denotes truncated CDR3 regions.

Supplementary Table 8. Clonotype counts of B7/NS1$_{196\text{-}206}$.

Clone ID	TRAV	TRAJ	CDR3α	CDR3α length	TRBV	TRBJ	CDR3β	CDR3 β length	KK102	BP146	BP155	BP184	BP185	BP189
A	TRAV38-2/DV8	TRAJ33	CAYRSAVGYQLIW	8	TRBV27	TRBJ1-5	CASSQYSNQPQHF	8	8					
B					TRBV27	TRBJ1-5	CASSQYSNQPQHF	8	5					
C					TRBV27	TRBJ1-1	CASSLMGNTEAFF	8	2	1				
D	TRAV38-2/DV8	TRAJ43	CAYRSAVTYDMRF	8	TRBV27	TRBJ1-5	CASSLYGNQPQHF	8	2					
E					TRBV28	TRBJ1-1	CASSLSRGWTEAFF	9	2					
F	TRAV6	TRAJ11	CAPLYSTLTF	5	TRBV27	TRBJ1-1	CASSLMGNTEAFF	8	1					
G	TRAV6	TRAJ29	CAPRNTPLVF	5	TRBV27	TRBJ1-5	CASSLYGGQPQHF	8	1					
H					TRBV27	TRBJ1-5	CASSLYGPGGVGQPQHF	12	1					
I	TRAV38-2/DV8	TRAJ33	CAYRSANNYQLIW	8	TRBV27	TRBJ2-1	CASSLYGSSYNEQFF	10	1					
J	TRAV3	TRAJ28	CAVRDPGAGSYQLTF	10	TRBV10-3	TRBJ2-7	CAISESGTGFEQYF	9	1					
K					TRBV11-2	TRBJ2-3	CASSLLTDTQYF	7	1					
L					TRBV14	TRBJ2-5	CASSQDFGRTQYF	8	1					
M	TRAV27	TRAJ34	CAGPPRPSYNTDKLIF	11	TRBV28	TRBJ1-2	CASTPRGRGLVYGYTF	11	1					
N	TRAV19	TRAJ13	CALSSTSGGYQKVTF	10	TRBV3-1	TRBJ1-1	CASSQERKAGGPTEAFF	12	1					
O	TRAV19	TRAJ4	CALGSFSGGYNKLIF	10	TRBV4-1	TRBJ2-5	CASSQLEQGGETQYF	10	1					
P	TRAV3	TRAJ6	CAVRDPSGGSYIPTF	10	TRBV5-8	TRBJ2-1	CASSPGSSGRLGEQFF	11	1					
Q	TRAV3	TRAJ28	CAVNSPGAGSYQLTF	10	TRBV7-3	TRBJ2-2	CASSSQGAAGELFF	9	1					
R	TRAV38-2/DV8	TRAJ44	CAYMSGTASKLTF	8					1					
S	TRAV19	TRAJ4	CALTRPGGGYNKLIF	10	TRBV2	TRBJ2-1	CASRGLQGRIDYNEQFF	12		4				
T					TRBV27	TRBJ2-7	CASRIYAGSPYEQYF	10		2				
U	TRAV38-2/DV8	TRAJ33	CAYRSASNYQLIW	8	TRBV27	TRBJ1-1	CASSLYPGANTEAFF	10		1				
V	TRAV38-2/DV8	TRAJ39	CAYRSASNAGNMLTF	10	TRBV27	TRBJ1-5	CASSLYRGSNQPQHF	10		1				
W					TRBV27	TRBJ1-6	CASSLYGAGNSPLHF	10		1				
X	TRAV38-2/DV8	TRAJ43	CAYRSSYNNNDMRF	9	TRBV27	TRBJ2-7	CASRIYAGSPYEQYF	10		1				
Y					TRBV27	TRBJ2-7	CASRLMPGSTYEQYF	10		1				
Z	TRAV10	TRAJ40	CVVSGTSGTYKYIF	9	TRBV19	TRBJ2-1	CASRARVLDEQFF	8		1				
AA	TRAV3	TRAJ8	CAVRDIMNTGFQKLVF	11	TRBV20-1	TRBJ2-1	CSARDSGLSHGEQFF	10		1				
AB					TRBV25-1	TRBJ2-2	CASSESGAGELFF	8		1				
AC					TRBV28	TRBJ1-2	CASNPAGGAGLGYGYTF	12		1				
AD	TRAV3	TRAJ23	CAVRDIRKIYNQGGKLIF	13	TRBV7-2	TRBJ2-5	CASSLGALGETQYF	9		1				
AE					TRBV7-9	TRBJ1-3	CASSLDSSSGNTIYF	10		1				
AF					TRBV7-9	TRBJ2-1	CASSLAGAGNEQFF	9		1				
AG	TRAV1-2	TRAJ28	CAVRLYSGAGSYQLTF	11	TRBV7-9	TRBJ2-7	CASSLDSSGGNEQYF	10		1				
AH					TRBV9	TRBJ1-5	CASSVGGAQPQHF	8		1				
AI					TRBV27	TRBJ2-1	CASSPHPGTSGGASYNEQFF	15			3			
AJ	TRAV38-2/DV8	TRAJ30	CAYRSAIQDKIIF	8	TRBV27	TRBJ2-1	CASSPHPGTSGGASYNEQFF	15			2			
AK	TRAV38-2/DV8	TRAJ41	CAYRSATGYALNF	8	TRBV27	TRBJ2-1	CASSLYGSVQNEQFF	10			2			
AL	TRAV3	TRAJ27	CAVRDIGTNAGKSTF	10	TRBV27	TRBJ2-2	CASSYRGLSGELFF	9			2			
AM	TRAV19	TRAJ13	CALSEYSGGYQKVTF	10	TRBV9	TRBJ2-1	CASSVATSGGSQQFF	10			2			
AN	TRAV38-2/DV8	TRAJ53	CAYRSAGSGGSNYKLTF	12	TRBV27	TRBJ1-5	CASSLMTNQPQHF	8			1			
AO	TRAV41	TRAJ26	CAVDNYGQNFVF	7	TRBV27	TRBJ2-3	CASSLYGADTQYF	8			1			
AP					TRBV4-1	TRBJ2-5	CASSQEVETQYF	7			1			
AQ	TRAV19	TRAJ13	CALRSDSGGYQKVTF	10	TRBV4-1	TRBJ2-5	CASSQEVETQYF	7			1			
AR					TRBV19	TRBJ2-4	CASSIVRGQRETKNIQYF	13			1			
AS	TRAV19	TRAJ4	CALGLFSGGYNKLIF	10	TRBV4-1	TRBJ2-5	CASSHRTGGETQYF	9			1			
AT					TRBV5-6	TRBJ2-6	CASSTILGPGANVLTF	11			1			
AU					TRBV7-9	TRBJ1-1	CASNGQGDTEAFF	8				2		
AV	TRAV3	TRAJ28	CAVRAPGAGSYQLTF	10								2		
AW	TRAV3	TRAJ28	CAVRDPGAGSYQLTF	10								2		
AX					TRBV11-2	TRBJ1-1	CASSPGSTEAFF	7				2		
AY					TRBV11-2	TRBJ2-2	CASSLGVEELFF	7				2		
AZ	TRAV3	TRAJ28	CAVRAPGAGSYQLTF	10	TRBV10-3	TRBJ2-7	CAISGQGASYEQYF	9				1		
BA	TRAV3	TRAJ28	CAVSGWGAGSYQLTF	10	TRBV7-9	TRBJ1-1	CASNGQGDTEAFF	8				1		

BB					TRBV10-1	TRBJ1-1	CASSESAGHKAFF	8				1		
BC	TRAV19	TRAJ13	CALTEYSGGYQKVTF	10	TRBV3-1	TRBJ1-4	CASSQDRDRGPDEKLFF	12				1		
BD	TRAV19	TRAJ13	CALSEKSGGYQKVTF	10	TRBV3-1	TRBJ2-3	CASSQERGDNTKNTQYF	12				1		
BE	TRAV3	TRAJ28	CAVGDPGAGSYQLTF	10	TRBV7-2	TRBJ2-7	CASSLPGTAYEQYF	9				1		
BF					TRBV7-9	TRBJ1-1	CASRGGRVAGADTEAFF	12				1		
BG	TRAV19	TRAJ4	CALLLFSGGYNKLIF	10	TRBV3-1	TRBJ2-5	CASSPREGGETQYF	9					7	
BH	TRAV14/DV4	TRAJ10	CAMREGPELTGGGNKLTF	13	TRBV20-1	TRBJ1-2	CSAKRNRAYHYGYTF	10					3	
BI					TRBV27	TRBJ1-5	CASSTLQGADQPQHF	10					1	
BJ					TRBV3-1	TRBJ2-5	CASSPREGGETQYF	9					1	
BK	TRAV1-2	TRAJ41	CAVRDRTPNSGYALNF	11	TRBV7-2	TRBJ1-1	CASSFGTGEAFF	7					1	
BL					TRBV7-2	TRBJ1-1	CASSFGTGEAFF	7					1	
BM					TRBV25-1	TRBJ2-1	CASSDGGSPQFF	7					1	
BN	TRAV19	TRAJ13	CALSRISGGYQKVTF	10	TRBV3-1	TRBJ1-4	CASSQERDRRAVEKLFF	12					1	
BO	TRAV38-2/DV8	TRAJ44	CAYRSGTASKLTF	8	TRBV4-3	TRBJ2-1	CASSQDRGLAGGTYNEQFF	14					1	
BP	TRAV25	TRAJ31	CAGRINNARLMF	7	TRBV6-5	TRBJ2-1	CASSRDLNEQFF	7					1	
BQ					TRBV7-9	TRBJ2-7	CASSPAGQGGYEQYF	10					1	
BR					TRBV9	TRBJ1-3	CASSVAGLGGNTIYF	10					1	
BS	TRAV16	TRAJ36	CALRSGANNLFF	7									1	
BT					TRBV14	TRBJ2-7	CASATSGTYYEQYF	9						2
BU	TRAV38-2/DV8	TRAJ29	CAYRSAIRNTPLVF	9	TRBV27	TRBJ1-5	CASSLYSNQPQHF	8						1
BV					TRBV27	TRBJ1-5	CASSFFGNQPQHF	8						1
BW					TRBV27	TRBJ1-5	CASSLMANQPQHF	8						1
BX	TRAV3	TRAJ28	CAVRALGAGSYQLTF	10	TRBV11-2	TRBJ1-1	CASSLDRVSVNTEAFF	11						1
BY	TRAV8-1	TRAJ13	CAVNDMNSGGYQKVTF	11	TRBV12-5	TRBJ2-1	CASGAPGTPYNEQFF	10						1
BZ					TRBV14	TRBJ1-6	CASSQIRETGSPLHF	10						1
CA	TRAV3	TRAJ28	CAVTVPGAGSYQLTF	10	TRBV14	TRBJ2-1	CASSGRGSYNEQFF	9						1
CB					TRBV14	TRBJ2-3	CASGQGALADTQYF	9						1
CC	TRAV14/DV4	TRAJ10	CAMREGPKLTGGGNKLTF	13	TRBV20-1	TRBJ2-1	CSANPRDESYNEQFF	10						1
CD	TRAV3	TRAJ28	CAVTAPGAGSYQLTF	10	TRBV20-1	TRBJ2-7	CSAPGGGGTSYEQYF	10						1
CE					TRBV4-1	TRBJ1-1	CASSQDRHTEAFF	8						1
CF	TRAV19	TRAJ4	CALLIFSGGYNKLIF	10	TRBV4-1	TRBJ2-5	CASSQEGGGETQYF	9						1
CG	TRAV3	TRAJ24	CAVRALTDSWGKLQF	10	TRBV4-3	TRBJ2-1	CASMARTSGDNEQFF	10						1
CH	TRAV13-2	TRAJ6	CAETPSGGSYIPTF	9	TRBV5-6	TRBJ2-7	CASSLGHGRSSYEQYF	11						1
CI					TRBV7-2	TRBJ2-5	CASSLPAGQETQYF	9						1
CJ	TRAV8-1	TRAJ39	CAVRVNNAGNMLTF	9	TRBV7-2	TRBJ2-5	CASSPGLPEYF	6						1
CK	TRAV3	TRAJ28	CASRDPGAGSYQLTF	10	TRBV7-9	TRBJ2-2	CASLEGYAAGELFF	9						1
CL	TRAV3	TRAJ40	CAVRDMASGTYKYIF	10	TRBV9	TRBJ1-1	CASSDRGMNTEAFF	9						1
CM	TRAV3	TRAJ42	CAVRAYYGGSQGNLIF	11	TRBV9	TRBJ1-2	CASSVDSGLGYGYTF	10						1
								Grand total	32	21	18	17	21	21

Red denotes truncated CDR3 regions.

Supplementary Table 9. Clonotype counts of B8/NP92-99.

Clone ID	TRAV	TRAJ	CDR3α	CDR3α length	TRBV	TRBJ	CDR3β	CDR3β length	BP140	BP147	BP155	BP170	BP185	BP191
A	TRAV17	TRAJ31	CATDESNARLMF	7	TRBV11-2	TRBJ2-7	CASSLIRTWDEQYF	9	3					
B					TRBV11-2	TRBJ2-7	CASSLIRTWDEQYF	9	2					
C	TRAV27	TRAJ47	SCGEGGNKLVF	6	TRBV27	TRBJ2-7	CASSWGQGKSSYEQYF	11	1					
D					TRBV3-1	TRBJ1-4	CASTSVRDRLEKLFF	10	1					
E					TRBV3-1	TRBJ2-3	CASSGSSGGRTDTQYF	11	1					
F					TRBV3-1	TRBJ2-7	CASSQARTAYEQYF	9	1					
G	TRAV1-2	TRAJ31	CAVRDWDNNARLMF	9					1					
H	TRAV29/DV5	TRAJ10	CAASVGGGNKLTF	8	TRBV3-1	TRBJ2-1	CASSPDRGYEQFF	8	1					
I	TRAV8-2	TRAJ11	CAVSGGYSTLTF	7	TRBV27	TRBJ2-7	CASSHRDRASGEGYEQYF	13	1					
J	TRAV8-2	TRAJ4	CVVSLGGYNKLIF	8	TRBV20-1	TRBJ2-1	CSARDRYNEQFF	7	1					
K					TRBV3-1	TRBJ2-5	CASSQWGRPQETQYF	10		3				
L	TRAV20	TRAJ45	CAVRTYSGGGADGLTF	11	TRBV12-3	TRBJ2-3	CASAPRRDRGTDTQYF	11		3				
M					TRBV9	TRBJ2-3	CASSPSELAGGRLDTQYF	13		2				
N	TRAV8-3	TRAJ20	CACGDYKLSF	5	TRBV9	TRBJ2-3	CASSPSELAGGRLDTQYF	13		2				
O	TRAV8-6	TRAJ32	CAVTNGGATNKLIF	9	TRBV3-1	TRBJ2-5	CASSQWGRPQETQYF	10		2				
P					TRBV11-2	TRBJ2-2	CASSLSRLAGDYTGELFF	13		1				
Q					TRBV27	TRBJ2-7	CASSIGQGKSSYEQYF	11		1				

R					TRBV9	TRBJ2-5	CASSVDPTGGGRQETQYF	13		1				
S	TRAV12-2	TRAJ40	CAVNPGTYKYIF	7	TRBV12-3	TRBJ2-3	CASAPRRDRGTDTQYF	11		1				
T	TRAV17	TRAJ54	CATDGRGGAQKLVF	9	TRBV3-1	TRBJ1-4	CASSSIAGRGEKLFF	10		1				
U	TRAV20	TRAJ45	CAVRTYSGGGADGLTF	11	TRBV12-3/TRBV12-4	TRBJ2-3	CASAPRRDRGTDTQYF	11		1				
V	TRAV22	TRAJ22	CAVEFPSSGSARQLTF	11	TRBV11-2	TRBJ2-3	CASSSPRVAGRPDTQYF	12		1				
W	TRAV26-1	TRAJ26	CIVRVAPSDDYGQNFVF	12	TRBV20-1	TRBJ1-1	CSARGDRGLNTEAFF	10		1				
X	TRAV26-1	TRAJ30	CIVRVAVSVSYRDKIIF	12						1				
Y	TRAV5	TRAJ37	CAETPIGSSNTGKLIF	11	TRBV25-1	TRBJ2-7	CASSVRYEQYF	6		1				
Z	TRAV8-3	TRAJ47	CAVGADGNKLVF	7	TRBV27	TRBJ2-7	CASSIGQGKSSYEQYF	11		1				
AA	TRAV29/DV5	TRAJ49	CAANTGNQFYF	6	TRBV11-2	TRBJ2-2	CASSWRTGNSGELFF	10			4			
AB					TRBV11-2	TRBJ2-2	CASSWRTGNSGELFF	10			3			
AC					TRBV11-1	TRBJ2-3	CASSLASGHRPTDTQYF	12			1			
AD					TRBV3-1	TRBJ2-3	CASSPRREQGDTQYF	10			1			
AE					TRBV4-1	TRBJ1-1	CASSLPPRNTEAFF	9			1			
AF	TRAV1-2	TRAJ11	CAVRPSGYSTLTF	8							1			
AG	TRAV20	TRAJ6	CAVRGSYIPTF	6	TRBV20-1	TRBJ1-1	CSAREGVNTEAFF	8			1			
AH	TRAV26-1	TRAJ26	CIVRVGPADNYGQNFVF	12	TRBV3-1	TRBJ2-3	CASSPRREQGDTQYF	10			1			
AI	TRAV29/DV5	TRAJ49	CAANTGNQFYF	6							1			
AJ	TRAV41	TRAJ49	CAVENTGNQFYF	7	TRBV4-1	TRBJ1-4	CASRAWGREGEKLFF	10			1			
AK	TRAV6	TRAJ38	CALPSNAGNNRKLIW	10	TRBV9	TRBJ2-2	CASSVERGRNTGELFF	11			1			
AL	TRAV3	TRAJ5	CAVAMDTGRRALTF	9								3		
AM	TRAV3	TRAJ5	CAVAMDTGRRALTF	9	TRBV9	TRBJ2-3	CASSNYPGGARTDTQYF	12				3		
AN	TRAV8-6	TRAJ52	CAVSLNAGGTSYGKLTF	12	TRBV18	TRBJ2-7	CASSPPRNMEPSYEQYF	12				3		
AO	TRAV19	TRAJ48	CALSEAPSNFGNEKLTF	12	TRBV4-1	TRBJ2-6	CASSHRDRGISGANVLTF	13				2		
AP	TRAV8-1	TRAJ13	CAVNMALGGGYQKVTF	11	TRBV5-6	TRBJ2-7	CASSPPARDRDHEQYF	11				2		
AQ	TRAV8-3	TRAJ20	CAVGDYKLSF	5	TRBV9	TRBJ2-3	CASSPSQTSGGRTDTQYF	13				2		
AR					TRBV5-6	TRBJ1-2	CASSLGDRGRGYGYTF	11				1		
AS	TRAV19	TRAJ48	CALSEAPSNFGNEKLTF	12								1		
AT	TRAV26-1	TRAJ41	CIVRVAASVSNSGYALNF	13	TRBV19	TRBJ1-1	CASSSRDRGLGTEAFF	11				1		
AU	TRAV3	TRAJ13	CAVRDLDSGGYQKVTF	11	TRBV16	TRBJ2-7	CASSHLDRGKKDYEQYF	12				1		
AV	TRAV8-3	TRAJ20	CAVGDYKLSF	5								1		
AW	TRAV4	TRAJ39	CLVGDSGNAGNMLTF	10	TRBV2	TRBJ1-4	CASRMTGGKTNEKLFF	11					8	
AX					TRBV2	TRBJ1-4	CASRMTGGKTNEKLFF	11					2	
AY	TRAV4	TRAJ45	CLVMDSGGGADGLTF	10									2	
AZ					TRBV27	TRBJ2-7	CASSWGQGKSSYEQYF	11					1	
BA					TRBV27	TRBJ2-7	CASSLGQGKSSYEQYF	11					1	
BB					TRBV3-1	TRBJ2-7	CASSPSRVLYEQYF	9					1	
BC					TRBV4-1	TRBJ2-1	CASSQDSGRGELFF	9					1	
BD					TRBV6-2	TRBJ1-1	CASTEPRGTEAFF	8					1	
BE	TRAV1-2	TRAJ15	CAVDQAGTALIF	7	TRBV18	TRBJ1-1	CASSPPRSEAFF	7					1	
BF	TRAV13	TRAJ26	CAASGNYGQNFVF	8	TRBV4-1	TRBJ1-4	CASSQDPPGRLGKLFF	11					1	
BG	TRAV20	TRAJ33	CAVDRGMDSNYQLIW	10	TRBV28	TRBJ2-7	CASSFLFRDGTYEQYF	11					1	
BH	TRAV22	TRAJ45	CAVVSWYSGGGADGLTF	12	TRBV9	TRBJ2-1	CASSPGDRGKTNEQFF	11					1	
BI	TRAV26-2	TRAJ49	CILRDVMNTGNQFYF	10	TRBV4-1	TRBJ2-7	CASSPRDRARGDEQYF	11					1	
BJ	TRAV29/DV5	TRAJ47	CAAMEYGNKLVF	7	TRBV9	TRBJ2-1	CASSVEIAGRYNEQFF	11					1	
BK	TRAV3	TRAJ28	CAVRQTAYSGAGSYQLTF	13	TRBV2	TRBJ2-3	CASSSSPRGDTQYF	9					1	
BL	TRAV36/DV7	TRAJ40	CAVSLPQEPTNTS	8	TRBV11-2	TRBJ2-1	CASTSRAGFNEQFF	9					1	
BM	TRAV8-2	TRAJ29	CVVSDPFGNTPLVF	9	TRBV29-1	TRBJ2-5	CSVWVGREQETQYF	9					1	
BN	TRAV9-2	TRAJ37	CAPLKGKLIF	5	TRBV11-2	TRBJ2-5	CASSLEGRREGETQYF	11					1	
BO	TRAV9-2	TRAJ39	CAQNAGNMLTF	6	TRBV20-1	TRBJ2-7	CSANPEGPYEQYF	8					1	
BP					TRBV28	TRBJ2-1	CASSLRLVAGRSYNEQFF	13						1
BQ					TRBV29-1	TRBJ2-3	CSVEGLGRAVTQYF	9						1
BR					TRBV4-1	TRBJ2-2	CASSQAGQAGELFF	9						1
BS					TRBV9	TRBJ1-1	CASSTRRNTEAFF	8						1
BT	TRAV12-2	TRAJ4	CAVNSGFSGGYNKLIF	11	TRBV11-2	TRBJ1-1	CASSFRQGEAFF	7						1
BU	TRAV21	TRAJ49	CAVLNTGNQFYF	7	TRBV12-3/TRBV12-4	TRBJ1-2	CASTVNRGSEGYTF	9						1
BV	TRAV27	TRAJ31	CAGEGDNNARLMF	8										1
BW	TRAV29/DV5	TRAJ26	CAARCHGQNFVF	7	TRBV3-1/TRBV3-2	TRBJ2-7	CASSYRSSYEQYF	8						1
BX	TRAV38-2/DV8	TRAJ33	CAYRSAARDSNYQLIW	11	TRBV12-5	TRBJ2-1	CASGLLTSGSINEQFF	11						1
								**Grand total**	**13**	**23**	**16**	**20**	**28**	**9**

Red denotes truncated CDR3 regions.

Supplementary Table 10. TCR sequencing primers

Primer Name	Sequence
huTRAV1ext	AACTGCACGTACCAGACATC
huTRAV2ext	GATGTGCACCAAGACTCC
huTRAV3ext	AAGATCAGGTCAACGTTGC
huTRAV4ext	CTCCATGGACTCATATGAAGG
huTRAV5ext	CTTTTCCTGAGTGTCCGAG
huTRAV6ext	CACCCTGACCTGCAACTATAC
huTRAV7ext	GCAAAATACAGGGATGGG
huTRAV8-1ext	CTCACTGGAGTTGGGATG
huTRAV8-3ext	CACTGTCTCTGAAGGAGCC
huTRAV8-2,4ext	GCCACCCTGGTTAAAGG
huTRAV8-6ext	GAGCTGAGGTGCAACTACTC
huTRAV8-7ext	CTAACAGAGGCCACCCAG
huTRAV9-1_2ext	TGGTATGTCCAATATCCTGG
huTRAV10ext	CAAGTGGAGCAGAGTCCTC
huTRAV12-1_3ext	CARTGTTCCAGAGGGAGC
huTRAV13-1ext	CATCCTTCAACCCTGAGTG
huTRAV13-2ext	CAGCGCCTCAGACTACTTC
huTRAV14ext	AAGATAACTCAAACCCAACCAG
huTRAV16ext	AGTGGAGCTGAAGTGCAAC
huTRAV17ext	GGAGAAGAGGATCCTCAGG
huTRAV18ext	TCCAGTATCTAAACAAAGAGCC
huTRAV19ext	AGGTAACTCAAGCGCAGAC
huTRAV20ext	CACAGTCAGCGGTTTAAGAG
huTRAV21ext	TTCCTGCAGCTCTGAGTG
huTRAV22ext	GTCCTCCAGACCTGATTCTC
huTRAV23ext	TGCTTATGAGAACACTGCG
huTRAV24ext	CTCAGTCACTGCATGTTCAG
huTRAV25ext	GGACTTCACCACGTACTGC
huTRAV26-1ext	GCAAACCTGCCTTGTAATC
huTRAV26-2ext	AGCCAAATTCAATGGAGAG
huTRAV27ext	TCAGTTTCTAAGCATCCAAGAG
huTRAV29ext	GCAAGTTAAGCAAAATTCACC
huTRAV30ext	CAACAACCAGTGCAGAGTC
huTRAV34ext	AGAACTGGAGCAGAGTCCTC
huTRAV35ext	GGTCAACAGCTGAATCAGAG
huTRAV36ext	GAAGACAAGGTGGTACAAAGC
huTRAV38ext	GCACATATGACACCAGTGAG
huTRAV39ext	CTGTTCCTGAGCATGCAG
huTRAV40ext	GCATCTGTGACTATGAACTGC
huTRAV41ext	AATGAAGTGGAGCAGAGTCC
huTRACext	GACCAGCTTGACATCACAG
huTRBV2ext	TCGATGATCAATTCTCAGTTG
huTRBV3ext	CAAAATACCTGGTCACACAG
huTRBV4ext	TCGCTTCTCACCTGAATG
huTRBV5-1_4ext	GATTCTCAGGKCKCCAGTTC
huTRBV5-5_8ext	GTACCAACAGGYCCTGGGT
huTRBV6-1_3,5_9ext	ACTCAGACCCCAAAATTCC

huTRBV6-4ext	ACTGGCAAAGGAGAAGTCC
huTRBV7-1_3ext	TRTGATCCAATTTCAGGTCA
huTRBV7-4_9ext	CGSWTCTYTGCAGARAGGC
huTRBV9ext	GATCACAGCAACTGGACAG
huTRBV10-1ext	CAGAGCCCAAGACACAAG
huTRBV10-2ext	ACCTTGATGTGTCACCAGAC
huTRBV11ext	CGATTTTCTGCAGAGACGC
huTRBV12ext	ARGTGACAGARATGGGACAA
huTRBV13ext	AGCGATAAAGGAAGCATCC
huTRBV14ext	CCAACAATCGATTCTTAGCTG
huTRBV15ext	AGTGACCCTGAGTTGTTCTC
huTRBV16ext	GTCTTTGATGAAACAGGTATGC
huTRBV17ext	CAGACCCCCAGACACAAG
huTRBV18ext	CATAGATGAGTCAGGAATGCC
huTRBV19ext	AGTTGTGAACAGAATTTGAACC
huTRBV20ext	AAGTTTCTCATCAACCATGC
huTRBV23ext	GCGATTCTCATCTCAATGC
huTRBV24ext	CCTACGGTTGATCTATTACTCC
huTRBV25ext	ACTACACCTCATCCACTATTCC
huTRBV27,28ext	TGGTATCGACAAGACCCAG
huTRBV29ext	TTCTGGTACCGTCAGCAAC
huTRBV30ext	TCCAGCTGCTCTTCTACTCC
huTRBCext	TAGAACTGGACTTGACAGCG
huTRAV1int	GCACCCACATTTCTKTCTTAC
huTRAV2int	CACTCTGTGTCCAATGCTTAC
huTRAV3int	ATGCACCTATTCAGTCTCTGG
huTRAV4int	ATTATATCACGTGGTACCAACAG
huTRAV5int	TACACAGACAGCTCCTCCAC
huTRAV6int	TGGTACCGACAAGATCCAG
huTRAV7int	TATGAGAAGCAGAAAGGAAGAC
huTRAV8-1int	GTCAACACCTTCAGCTTCTC
huTRAV8-3int	TTTGAGGCTGAATTTAAGAGG
huTRAV8-2,4int	AGAGTGAAACCTCCTTCCAC
huTRAV8-6int	AACCAAGGACTCCAGCTTC
huTRAV8-7int	ATCAGAGGTTTTGAGGCTG
huTRAV9-1_2int	GAAACCACTTCTTTCCACTTG
huTRAV10int	GAAAGAACTGCACTCTTCAATG
huTRAV12-1_3int	AAGATGGAAGGTTTACAGCAC
huTRAV13-1int	TCAGACAGTGCCTCAAACTAC
huTRAV13-2int	CAGTGAAACATCTCTCTCTGC
huTRAV14int	AGGCTGTGACTCTGGACTG
huTRAV16int	GTCCAGTACTCCAGACAACG
huTRAV17int	CCACCATGAACTGCAGTTAC
huTRAV18int	TGACAGTTCCTTCCACCTG
huTRAV19int	TGTGACCTTGGACTGTGTG
huTRAV20int	TCTGGTATAGGCAAGATCCTG
huTRAV21int	AACTTGGTTCTCAACTGCAG
huTRAV22int	CTGACTCTGTGAACAATTTGC

huTRAV23int	TGCATTATTGATAGCCATACG
huTRAV24int	TGCCTTACACTGGTACAGATG
huTRAV25int	TATAAGCAAAGGCCTGGTG
huTRAV26-1int	CGACAGATTCACTCCCAG
huTRAV26-2int	TTCACTTGCCTTGTAACCAC
huTRAV27int	CTCACTGTGTACTGCAACTCC
huTRAV29int	CTGCTGAAGGTCCTACATTC
huTRAV30int	AGAAGCATGGTGAAGCAC
huTRAV34int	ATCTCACCATAAACTGCACG
huTRAV35int	ACCTGGCTATGGTACAAGC
huTRAV36int	ATCTCTGGTTGTCCACGAG
huTRAV38int	CAGCAGGCAGATGATTCTC
huTRAV39int	TCAACCACTTCAGACAGACTG
huTRAV40int	GGAGGCGGAAATATTAAAGAC
huTRAV41int	TTGTTTATGCTGAGCTCAGG
huTRACint	TGTTGCTCTTGAAGTCCATAG
huTRBV2int	TTCACTCTGAAGATCCGGTC
huTRBV3int	AATCTTCACATCAATTCCCTG
huTRBV4int	CCTGCAGCCAGAAGACTC
huTRBV5-1_4int	CTTGGAGCTGGRSGACTC
huTRBV5-5_8int	TCTGAGCTGAATGTGAACG
huTRBV6-1_3,5_9int	GTGTRCCCAGGATATGAACC
huTRBV6-4int	TGGTTATAGTGTCTCCAGAGC
huTRBV7-1_3int	TCYACTCTGAMGWTCCAGCG
huTRBV7-4_9int	TGRMGATYCAGCGCACA
huTRBV9int	GTACCAACAGAGCCTGGAC
huTRBV10-1int	TGGTATCGACAAGACCTGG
huTRBV10-3int	GGAACACCAGTGACTCTGAG
huTRBV11int	GACTCCACTCTCAAGATCCA
huTRBV12int	CYACTCTGARGATCCAGCC
huTRBV13int	CATTCTGAACTGAACATGAGC
huTRBV14int	ATTCTACTCTGAAGGTGCAGC
huTRBV15int	ATAACTTCCAATCCAGGAGG
huTRBV16int1	CTGTAGCCTTGAGATCCAGG
huTRBV17int	TGTTCACTGGTACCGACAG
huTRBV18int	CGATTTTCTGCTGAATTTCC
huTRBV19int	TTCCTCTCACTGTGACATCG
huTRBV20int	ACTCTGACAGTGACCAGTGC
huTRBV23int	GCAATCCTGTCCTCAGAAC
huTRBV24int	GATGGATACAGTGTCTCTCGA
huTRBV25int	CAGAGAAGGGAGATCTTTCC
huTRBV27,28int	TTCYCCCTGATYCTGGAGTC
huTRBV29int	TCTGACTGTGAGCAACATGAG
huTRBV30int	AGAATCTCTCAGCCTCCAGAC
huTRBCint	TTCTGATGGCTCAAACACAG
