# Supplementary material for: CD8+ T-cell responses towards conserved influenza B virus epitopes across anatomical sites and age
Source: Nat Commun. 2024 Apr 29;15:3387. doi: 10.1038/s41467-024-47576-y (PMC11059233; doi:10.1038/s41467-024-47576-y)
Supplement: Supplementary file 3 — Description of Additional Supplementary Files [file 41467_2024_47576_MOESM3_ESM.pdf]

Description of Additional Supplementary Files

File Name: Supplementary Data 1

Description: Immunopeptidomics peptide identification File contains influenza B virus (IBV) peptides identified through immunopeptidomics and their predicted binding affinities to HLA-B*07:02, HLA-B*08:01, HLA-B*35:01, HLA-B*35:03 and HLAC*04:01.

File Name: Supplementary Data 2

Description: List of influenza B viruses obtained from NCBI Influenza Virus Resource database accession numbers File contains list of NCBI accession numbers for influenza B (IBV) sequences used to analyse sequence conservation within IBV proteins.

(https://www.ncbi.nlm.nih.gov/genomes/FLU/Database/nph-select.cgi?go=database)
