# Supplementary material for: CD8+ T-cell responses towards conserved influenza B virus epitopes across anatomical sites and age
Source: Nat Commun. 2024 Apr 29;15:3387. doi: 10.1038/s41467-024-47576-y (PMC11059233; doi:10.1038/s41467-024-47576-y)
Supplement: Supplementary file 6 — Reporting Summary [file 41467_2024_47576_MOESM6_ESM.pdf]

Corresponding author(s): Katherine Kedzierska
Last updated by author(s): 2024/02/01

Reporting Summary

Nature Portfolio wishes to improve the reproducibility of the work that we publish. This form provides structure for consistency and transparency in reporting. For further information on Nature Portfolio policies, see our Editorial Policies and the Editorial Policy Checklist.

Statistics

For all statistical analyses, confirm that the following items are present in the figure legend, table legend, main text, or Methods section.

n/a	Confirmed	
☐	☒	The exact sample size (*n*) for each experimental group/condition, given as a discrete number and unit of measurement
☐	☒	A statement on whether measurements were taken from distinct samples or whether the same sample was measured repeatedly
☐	☒	The statistical test(s) used AND whether they are one- or two-sided<br>*Only common tests should be described solely by name; describe more complex techniques in the Methods section.*
☐	☒	A description of all covariates tested
☐	☒	A description of any assumptions or corrections, such as tests of normality and adjustment for multiple comparisons
☐	☒	A full description of the statistical parameters including central tendency (e.g. means) or other basic estimates (e.g. regression coefficient) AND variation (e.g. standard deviation) or associated estimates of uncertainty (e.g. confidence intervals)
☐	☒	For null hypothesis testing, the test statistic (e.g. *F*, *t*, *r*) with confidence intervals, effect sizes, degrees of freedom and *P* value noted<br>*Give P values as exact values whenever suitable.*
☒	☐	For Bayesian analysis, information on the choice of priors and Markov chain Monte Carlo settings
☒	☐	For hierarchical and complex designs, identification of the appropriate level for tests and full reporting of outcomes
☒	☐	Estimates of effect sizes (e.g. Cohen's *d*, Pearson's *r*), indicating how they were calculated

Our web collection on statistics for biologists contains articles on many of the points above.

Software and code

Policy information about availability of computer code

Data collection	BD FSR Fortessa, BD FACS Aria III
Data analysis	Flowjo v10.8.1, GraphPad Prism v9.5.1, R v4.1.3, TCRdist, Finch TV v1.4.0

For manuscripts utilizing custom algorithms or software that are central to the research but not yet described in published literature, software must be made available to editors and reviewers. We strongly encourage code deposition in a community repository (e.g. GitHub). See the Nature Portfolio guidelines for submitting code & software for further information.

Data

Policy information about availability of data

All manuscripts must include a data availability statement. This statement should provide the following information, where applicable:

- Accession codes, unique identifiers, or web links for publicly available datasets
- A description of any restrictions on data availability
- For clinical datasets or third party data, please ensure that the statement adheres to our policy

Data will be made available according to our data availability statement provided in the manuscript:
Data availability. TCR sequence data (Supplementary Table 5-8, Source data) has been deposited in Mendeley [https://doi.org/10.17632/wg7swwf6jr.1] and VDJdb (https://vdjdb.cdr3.net). Mass spectrometry proteomics data have been deposited in the ProteomeXchange Consortium via the PRIDE83 partner repository with the dataset identifier PXD045000 [https://doi.org/10.6019/PXD045000]. Crystal structure have been deposited into the RSCB protein data bank (8TUH; HLA-B*07:02-

NP30-38 and 8TUB; HLA-B*07:02-NS1196-206). Any additional information needed to reanalyze the data in this paper is available from the corresponding author upon reasonable request. Source data are provided in this paper.

Other data sets utilised in this manuscript are IEDB and UniProt

Specific details:
The TCR data will be available on Mendely and VDJdb following publication. The Mendley doi has been reserved and the data will be accessible after publication. VDJdb data is linked to PMID and not with a doi.

Research involving human participants, their data, or biological material

Policy information about studies with human participants or human data. See also policy information about sex, gender (identity/presentation), and sexual orientation and race, ethnicity and racism.

Reporting on sex and gender	Please refer to Supplementary Table 2
Reporting on race, ethnicity, or other socially relevant groupings	We also reported the age groups of our donors but not other socially relevant groupings.
Population characteristics	Please refer to Supplementary Table 2
Recruitment	Samples were recruited via the University of Melbourne (UoM; Melbourne, Australia), Deepdene Medical Clinic (DMC; Deepdene, Australia via co-author J. Crowe), Australian Red Cross Lifeblood (ARCL; Melbourne, Australia), Launceston General Hospital (Tasmania, Australia, via co-author K. Flanagan), the Alfred Hospital's Lung Tissue Biobank and DonateLife Victoria. All donors were recruited randomly and on a voluntary basis.<br>Signed informed consent was obtained from all donors or their guardians prior to the study. Participants did not receive compensation.
Ethics oversight	Experiments were conducted in accordance with the Declaration of Helsinki Principles and according to the Australian National Health and Medical Research Council Code of Practice. Informed consent was obtained from all donors or their guardians prior to the study. The study was approved by the Human Research Ethics Committee (HREC) of the University of Melbourne Experiments (Ethics ID 13344, 24567), Australian Red Cross Lifeblood (ID 2015#8) and Tasmanian Health and Medical HREC (ID H0017479).

Note that full information on the approval of the study protocol must also be provided in the manuscript.

Field-specific reporting

Please select the one below that is the best fit for your research. If you are not sure, read the appropriate sections before making your selection.

☒ Life sciences ☐ Behavioural & social sciences ☐ Ecological, evolutionary & environmental sciences

For a reference copy of the document with all sections, see nature.com/documents/nr-reporting-summary-flat.pdf

Life sciences study design

All studies must disclose on these points even when the disclosure is negative.

Sample size	Sample size was determined by sample availability
Data exclusions	No data were excluded with the following exception: Donors who had a total number of less then 10 counted tetramer+CD8+ T cells within the whole enriched fraction were excluded for further phenotypic analysis as cell numbers were to low. This was indicated in the manuscript.
Replication	Experiments with human PBMCs could not be replicated due to limited PBMC numbers. For immunopeptidomic experiments the number of replicates were as follows:<br>C1R.B*07:02 - 1 uninfected, 2 B/Malaysia infected<br>C1R.B*08:01 - 3 uninfected, 3 B/Malaysia infected<br>C1R.B*35:01 - 1 uninfected, 2 B/Malaysia infected.<br><br>All attempts at replication were successful.
Randomization	N/A. PBMC donors were chosen from a large randomly recruited and HLA typed cohort of n = ~500 consisting of 102 children, 324 adults and 75 elderly donors based on their expression of HLA-B*07:02, HLA-B*08:01 and HLA-B*35:01. Samples were used based on availability.<br><br>Randomization was not applicable for immunopeptidomics experiments, as they utilised cell lines, and was not applicable for crystal

	structures.
Blinding	Experiments were not blinded as donors with the relevant HLA alleles needed to be chosen. Instead all analyses have been independently checked by multiple researchers.

Reporting for specific materials, systems and methods

We require information from authors about some types of materials, experimental systems and methods used in many studies. Here, indicate whether each material, system or method listed is relevant to your study. If you are not sure if a list item applies to your research, read the appropriate section before selecting a response.

Materials & experimental systems

n/a	Involved in the study	
☐	☒	Antibodies
☐	☒	Eukaryotic cell lines
☒	☐	Palaeontology and archaeology
☒	☐	Animals and other organisms
☒	☐	Clinical data
☒	☐	Dual use research of concern
☒	☐	Plants

Methods

n/a	Involved in the study	
☒	☐	ChIP-seq
☐	☒	Flow cytometry
☒	☐	MRI-based neuroimaging

Antibodies

Antibodies used

We used commercially-available antibodies as per Materials and Methods:
ICS:
(Surface staining) anti-CD107a-FITC (1:200, Invitrogen #53-1079-42), Live/Dead near infrared (NIR) (1:800, Invitrogen #L34976), anti-CD8-PerCP-Cy5.5 (1:00, BD Pharmingen #565310), anti-CD4-PE (1:50, BD Pharmingen #555347) or anti-CD4-BV650 (1:200, BD Horizon #563875) and anti-CD3-PE-Cy7 (1:50, BD Pharmingen #563423).

(Intracellular): anti-IFN--V450 (1:100, BD Pharmingen #560371), anti-MIP-1β-APC (1:40, BD Pharmingen #560686) or anti-MIP-1β-PE (1:50, BD Pharmingen #550078), anti-TNFα-AF700 (1:50, BD Pharmingen #557996)

Tetramer staining of cell lines:
anti-CD3-BV510 (1:200, BioLegend #317332), anti-CD4-BV650 (1:200, BD Horizon #563875), Live/Dead NIR (1:800, Invitrogen #L34976) and anti-CD8-PerCP-Cy5.5 (1:50, BD Pharmingen #565310)

PBMC TAMES: anti-CD71-BV421 (1:50, BD Horizon #562995), anti-CD3-BV510 (1:200, BioLegend #317332), anti-HLA-DR-BV605 (1:100, BioLegend #307640), anti-CD4-BV650 (1:200, BD Horizon #563875), anti-CD27-BV711 (1:200, BD Horizon #563167), anti-CD38-BV786 (1:100, BD Horizon #563964), anti-CCR7-AF700 (1:50, BD Pharmingen #561143), anti-CD14-APC-H7 (1:100, BD Pharmingen #560180), anti-CD19-APC-H (1:100, BD Pharmingen #560177 or #560252), anti-Live/Dead NIR (1:800, Invitrogen #L34976), anti-CD45RA-FITC (1:200, BD Pharmingen #555488), anti-CD8-PerCP-Cy5.5 (1:50, BD Pharmingen #565310), anti-CD95-PE-CF594 (1:100, BD Horizon #562395) and anti-PD-1-PE-Cy7 (1:50, BD Pharmingen #561272).

Tissue TAMES: anti-CD103-BUV395 (1:50, BD Horizon #564346), anti-CD69-BV421 (BioLegend #310930), anti-CD3-BV510 (1:200, BioLegend #317332), anti-HLA-DR-BV605 (1:100, BioLegend #307640), anti-CD4-BV650 (1:200, BD Horizon #563875), anti-CD27-BV711 (1:200, BD Horizon #563167), anti-CD38-BV786 (1:100, BD Horizon #563964), anti-CCR7-AF700 (1:50, BD Pharmingen #561143), anti-CD14-APC-H7 (1:100, BD Pharmingen #560180), anti-CD19-APC-H (1:100, BD Pharmingen #560177 or #560252), anti-Live/Dead NIR (1:800, Invitrogen #L34976), anti-CD45RA-FITC (1:200, BD Pharmingen #555488), anti-CD8-PerCP-Cy5.5 (1:50, BD Pharmingen #565310), anti-CD95-PE-CF594 (1:100, BD Horizon #562395) and anti-PD-1-PE-Cy7 (1:50, BD Pharmingen #561272).

Validation

All antibodies were validated for use on human cells by the manufacturers, and were titrated in our laboratory before use.

Eukaryotic cell lines

Policy information about cell lines and Sex and Gender in Research

Cell line source(s)	C1R cell lines were obtained from the Chen laboratory (La Trobe University), C1R.B*07:02 and C1R.B*35:01 cell lines were obtained from the Department of Biochemistry and Molecular Biology & Infection and Immunity Program, Biomedicine Discovery Institute Monash University , and C1R.B*08:01 cell lines were obtained from the McCLuskey Laboratory (University of Melbourne). MDCK cells were obtained from the American Type Culture Collection.
Authentication	Cell lines were not formally authenticated. C1R.HLA cells were routinely tested for HLA expression level prior to each experiment.

Mycoplasma contamination	Cell lines tested negative for mycoplasma contamination.
Commonly misidentified lines (See ICLAC register)	None were used in this study.

Plants

Seed stocks	*Report on the source of all seed stocks or other plant material used. If applicable, state the seed stock centre and catalogue number. If plant specimens were collected from the field, describe the collection location, date and sampling procedures.*
Novel plant genotypes	*Describe the methods by which all novel plant genotypes were produced. This includes those generated by transgenic approaches, gene editing, chemical/radiation-based mutagenesis and hybridization. For transgenic lines, describe the transformation method, the number of independent lines analyzed and the generation upon which experiments were performed. For gene-edited lines, describe the editor used, the endogenous sequence targeted for editing, the targeting guide RNA sequence (if applicable) and how the editor was applied.*
Authentication	*Describe any authentication procedures for each seed stock used or novel genotype generated. Describe any experiments used to assess the effect of a mutation and, where applicable, how potential secondary effects (e.g. second site T-DNA insertions, mosiacism, off-target gene editing) were examined.*

Flow Cytometry

Plots

Confirm that:

- [x] The axis labels state the marker and fluorochrome used (e.g. CD4-FITC).
- [x] The axis scales are clearly visible. Include numbers along axes only for bottom left plot of group (a 'group' is an analysis of identical markers).
- [x] All plots are contour plots with outliers or pseudocolor plots.
- [x] A numerical value for number of cells or percentage (with statistics) is provided.

Methodology

Sample preparation	Samples were prepared as described in Methods
Instrument	BD LSRII Fortessa and BD FACSAriaIII were used for acquisition of data, BD FACS Aria III was used for single cell index sorting.
Software	BD FACS Diva, FlowJo
Cell population abundance	Only single cell sorting was performed, which was confirmed by the presence of single TCR chains.
Gating strategy	Gating strategy has been described in fig .1e, fig. 3a, fig. 4a, fig. 5a and c, fig. 6a, fig. 7a, fig. 9a, sup fig. 1a, sup fig. 2a, sup fig. 3a, sup fig. 5a

- [x] Tick this box to confirm that a figure exemplifying the gating strategy is provided in the Supplementary Information.
